# Effector gene reshuffling involves dispensable mini-chromosomes in the wheat blast fungus

**DOI:** 10.1371/journal.pgen.1008272

**Published:** 2019-09-12

**Authors:** Zhao Peng, Ely Oliveira-Garcia, Guifang Lin, Ying Hu, Melinda Dalby, Pierre Migeon, Haibao Tang, Mark Farman, David Cook, Frank F. White, Barbara Valent, Sanzhen Liu

**Affiliations:** 1 Department of Plant Pathology, Kansas State University, Manhattan, KS, United States of America; 2 Department of Plant Pathology, University of Florida, Gainesville, FL, United States of America; 3 Center for Genomics and Biotechnology and Fujian Provincial Key Laboratory of Haixia Applied Plant Systems Biology, Fujian Agriculture and Forestry University, Fujian, China; 4 Department of Plant Pathology, University of Kentucky, Lexington, KY, United States of America; University of Georgia, UNITED STATES

## Abstract

Newly emerged wheat blast disease is a serious threat to global wheat production. Wheat blast is caused by a distinct, exceptionally diverse lineage of the fungus causing rice blast disease. Through sequencing a recent field isolate, we report a reference genome that includes seven core chromosomes and mini-chromosome sequences that harbor effector genes normally found on ends of core chromosomes in other strains. No mini-chromosomes were observed in an early field strain, and at least two from another isolate each contain different effector genes and core chromosome end sequences. The mini-chromosome is enriched in transposons occurring most frequently at core chromosome ends. Additionally, transposons in mini-chromosomes lack the characteristic signature for inactivation by repeat-induced point (RIP) mutation genome defenses. Our results, collectively, indicate that dispensable mini-chromosomes and core chromosomes undergo divergent evolutionary trajectories, and mini-chromosomes and core chromosome ends are coupled as a mobile, fast-evolving effector compartment in the wheat pathogen genome.

## Author summary

The emerging blast disease on wheat is proving even harder to control than the ancient, still-problematic rice blast disease. Potential wheat resistance identified using strains isolated soon after disease emergence are no longer effective in controlling recent aggressive field isolates from wheat in South America and South Asia. We construct a high-quality assembly of an aggressive, recently-isolated wheat blast fungal strain and the first assembled mini-chromosome genome sequence of wheat and rice blast pathogens. We report that recent wheat pathogens can contain one or two highly-variable dispensable mini-chromosomes, each with an amalgamation of fungal effector genes and other sequences that are duplicated or absent from indispensable core chromosome ends. Well-studied effectors found on different core chromosomes in rice pathogens appear side-by-side in wheat pathogen mini-chromosomes. The rice pathogen often overcomes deployed resistance genes by deleting triggering effector genes. We propose that the fast-evolving effector-rich compartment of the wheat blast fungus is a combination of core chromosome ends and mobile mini-chromosomes that are easily lost from individual strains. Localization of effectors on mini-chromosomes would therefore accelerate pathogen adaptation in the field.

## Introduction

**Fig 1:**
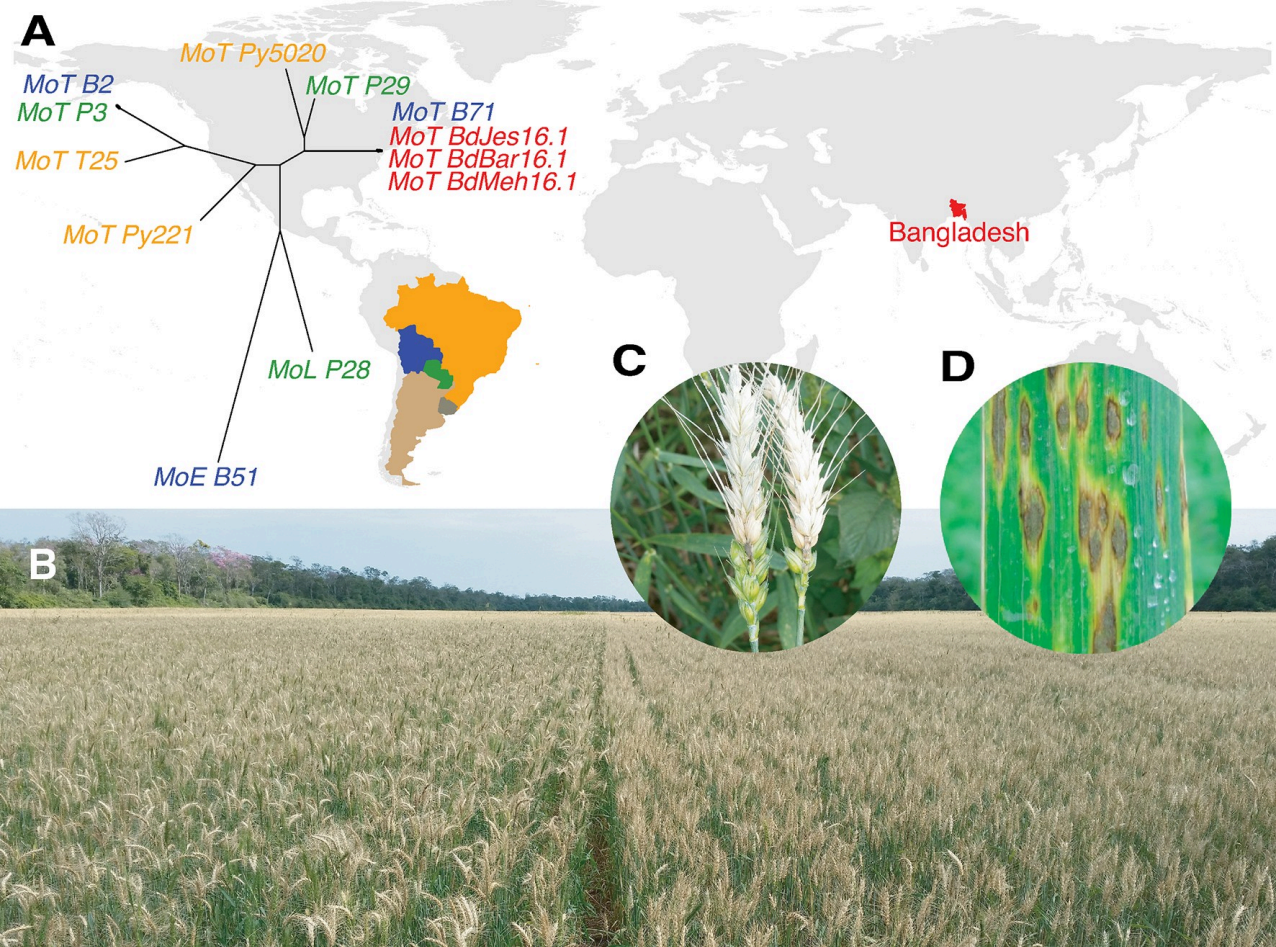
Wheat blast disease has now spread from South America and established in South Asia. **(A)** Countries with wheat blast are labeled with non-gray colors. The phylogeny contains nine strains examined in this study and three from Bangladesh. Strain names are color-coded with country colors on the map and they indicate the *M*. *oryzae* lineage represented: MoT for the *Triticum* lineage; MoL for the *Lolium* lineage and MoE for the *Eleusine* lineage. See also **S1 Fig**. **(B)** A wheat field in Bolivia in 2015 shows near 100% killed (straw-colored) heads. The field appeared healthy before heading. **(C)** Close up of infected wheat heads with spikelets removed to show fungus at the point of infection on the stem. **(D)** Sporulating leaf lesions, sometimes found on highly susceptible wheat varieties.

Wheat blast is an explosive emerging disease capable of 100% yield losses. Little resistance is available in cultivated wheat varieties, and fungicides are not effective under disease favorable conditions [1,2]. The disease emerged in Brazil in 1985 and spread within South America, limiting wheat production **(Fig 1)**. Wheat blast jumped continents in 2016, causing major yield losses in Bangladesh with this first report [3,4]. Wheat blast has now established in South Asia, enhancing fears about further disease spread, disruption of global grain trade by this seed-borne pathogen, and endangerment of global food security [5]. Wheat blast is caused by a wheat-adapted lineage of *Magnaporthe oryzae* (synonymous with *Pyricularia oryzae*) [6], known as the *Triticum* pathotype (MoT). MoT strains are distinct from rice pathogens in the *M*. *oryzae Oryza* pathotype (MoO) and millet pathogens in the *Eleusine* (MoE) and *Setaria* (MoS) pathotypes (**S1 Fig**). A serious turf grass disease emerged in the United States in the late 1980s, caused by the *Lolium* pathotype (MoL) with ryegrass as its major host. Although some MoL strains can infect wheat [7], MoT strains are distinguished as highly aggressive wheat pathogens that are so far restricted to certain countries in South America and South Asia (**Fig 1A**).

Although little is known about wheat blast, studies on rice blast disease have identified numerous effector genes, generally encoding small proteins that are specifically expressed *in planta* and play roles in host invasion [8–10]. Some effectors, termed avirulence (AVR) effectors, determine either rice cultivar or host species specificity through blocking infection upon recognition by corresponding cultivar- or species-specific resistance (*R*) genes and triggering hypersensitive resistance. For example, strains of several *M*. *oryzae* pathotypes are not able to infect weeping lovegrass, *Eragrostis curvula*, because they carry a host species-specific *AVR* effector *PWL2* [11,12]. Planting of wheat varieties lacking the *R* gene *Rwt3* in Brazil likely enabled MoL strains with the corresponding host species-specific *AVR* effector *PWT3* to adapt to wheat, and subsequent loss of *PWT3* function played a role in the wider emergence of the MoT subgroup [13]. So far, characterization of 11 MoO *AVR* effectors together with their corresponding *R* gene products has identified direct or indirect protein interactions that control rice cultivar specificity [9,14]. In contrast, understanding how individual effectors function in host invasion has been difficult due to apparent functional redundancy. That is, deletion of individual effector genes rarely dramatically impacts the pathogen's ability to cause disease.

Effector genes in diverse filamentous eukaryotic pathogens generally reside in rapidly evolving, transposon-rich chromosomal regions, which, together with slowly evolving core chromosome regions containing housekeeping genes, results in a 'two-speed' genome [15,16]. *M*. *oryzae* effectors from the *Oryza* pathotype are known to reside in transposon-rich regions, often near chromosome ends [9,17]. Two *AVR* effector genes [18,19] have been localized to dispensable mini-chromosomes (also known as supernumerary, accessory or B chromosomes [16,20,21]) that show non-mendelian inheritance and are present in some, but not all individuals in a population [22–24]. Effectors are associated with frequent presence/absence polymorphisms between and/or within the different *M*. *oryzae* lineages [18,25]. Deletion of the corresponding *AVR* effector gene could be a response to deploying *R* genes in a crop. In one well-studied case, *AVR-Pita1*, which corresponds to the periodically-deployed *Pita* rice *R* gene, has been mobile in the *M*. *oryzae* genome [18]. Specifically, *AVR-Pita1* is found on different chromosomes in different strains, often near telomeres, and sometimes on mini-chromosomes. Understanding *AVR* effector gene dynamics is key to combating the ability of the blast fungus to rapidly overcome deployed *R* genes and to developing sustainable disease control.

Wheat blast disease is proving even harder to control than the ancient, still-problematic rice blast disease. Potential wheat resistance identified using strains isolated soon after disease emergence in 1985 are no longer effective in controlling recent aggressive field isolates from wheat in South America and South Asia. The global threat now posed by wheat blast disease makes it critical to generate genomic resources to further understand the wheat blast fungus. Here, a reference genome of an aggressive MoT strain was generated and compared to genomes of early and recent wheat pathogens and other host-adapted strains. We report that the genome structures of the 7 wheat blast core chromosomes have not diverged significantly from the rice blast core chromosomes. However, mini-chromosomes present in zero, one or two copies in different strains serve as a highly variable compartment for effector genes.

## Results

### A reference genome of the MoT strain B71

We sequenced and generated a near-complete genome assembly of the highly aggressive Bolivian field isolate B71 [4,26], which exhibits high sequence similarity with MoT isolates from Bangladesh (**Fig 1A, S1 Table and S1 Fig**). An assemblage containing 31 contigs (**S2 Table**) was produced from >12.4 Gb of whole genome shotgun (WGS) PacBio long reads (**S2 Fig**). Genome polishing utilizing ~10 Gb Illumina sequencing data corrected 37,982 small insertions and deletions as well as 350 base-pair substitutions in the PacBio draft assembly (**S1 Data**). Corrected assembled contigs were in the range of 44.2% to 52.5% GC content with the exception of a contig of 28.4%, which was predicted to be from mitochondria of B71 owing to its high similarity (99% identity) to the mitochondrial sequence of *M*. *oryzae* rice pathogen 70–15 [27]. A circularized B71 mitochondrial sequence was obtained after removing redundant sequences at the contig ends.

**Fig 2:**
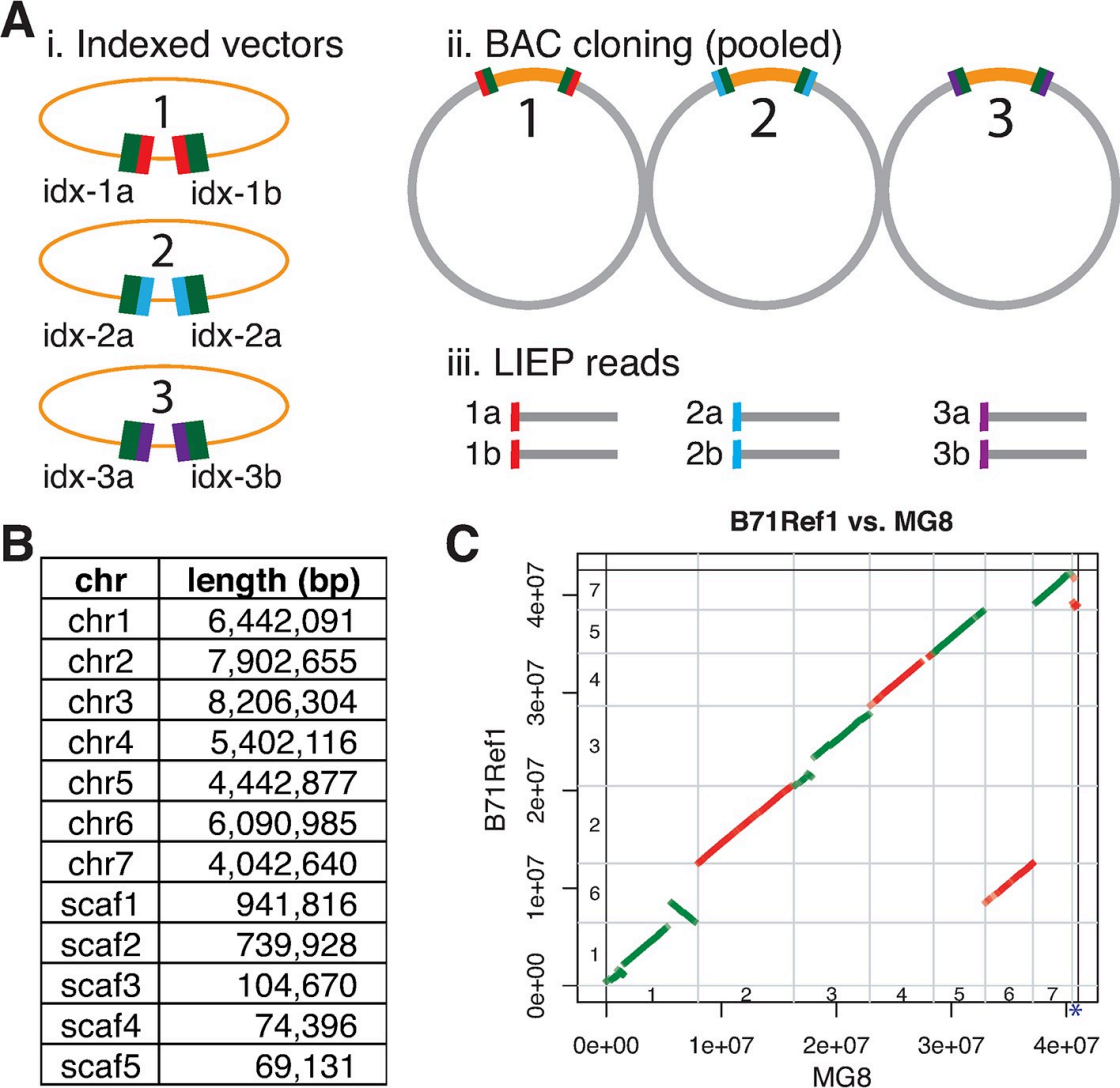
LIEP procedure and B71 assembly. **(A)** Each vector molecule contains two Illumina adaptor sequences (green) and random barcodes. The pool of barcoded vectors were pre-sequenced and are used to construct a clone library. Clone-ends, each of which included a barcode, are sequenced massively and assembled separately. Paired clone-end sequences (e.g., idx-1a and idx-1b) from the same clone are identified based on barcodes. **(B)** Lengths of 12 sequences of the B71 assembly. **(C)** A dotplot to compare the collinearity between the B71 assembly and MG8, the assembly for rice strain 70–15. Alignments between the two assemblies were performed by using Nucmer. Only alignments with at least 10 kb match and at least 95% identity were shown. Chromosomes numbers, 1–7, were indicated inside axes. The blue highlighted asterisk represents the unanchored MG8 contig (supercont8.8) that was mapped at the beginning of B71 chromosome 7. Accumulative chromosomal positions were labeled on both x- and y-axis.

We developed a novel scaffolding technology, LIEP (Long Insert End-Pair sequencing) to improve the continuity of the assembly (**Fig 2A**). Briefly, LIEP involved construction of millions of vectors, each of which contains a unique DNA barcode pair of 22 nt and 21 nt random barcodes. Barcodes for each vector were sequenced to establish a sequence database of barcode pairs. The vectors were then used to construct clones with 20–30 kb long inserts of B71 genomic DNA flanked by the two vector barcodes. Both ends of the insert were sequenced, generating clone-end sequences with paired barcode sequences. Barcode sequences were used to recover clone-end pairs. All steps were performed with pooled clones rather than individual clones. After scaffolding, a small contig (~12 kb) with the poor support from Illumina reads was discarded. Scaffolding and filtering condensed the assembly to 12 contigs, which were then reoriented and renamed based on the MG8 genome assembly of rice pathogen 70–15 [27]. Consequently, the final B71 genome assembly (B71Ref1) is comprised of ~44.46 Mb in seven chromosomes and five unanchored scaffolds (**Fig 2B**).

**Fig 3:**
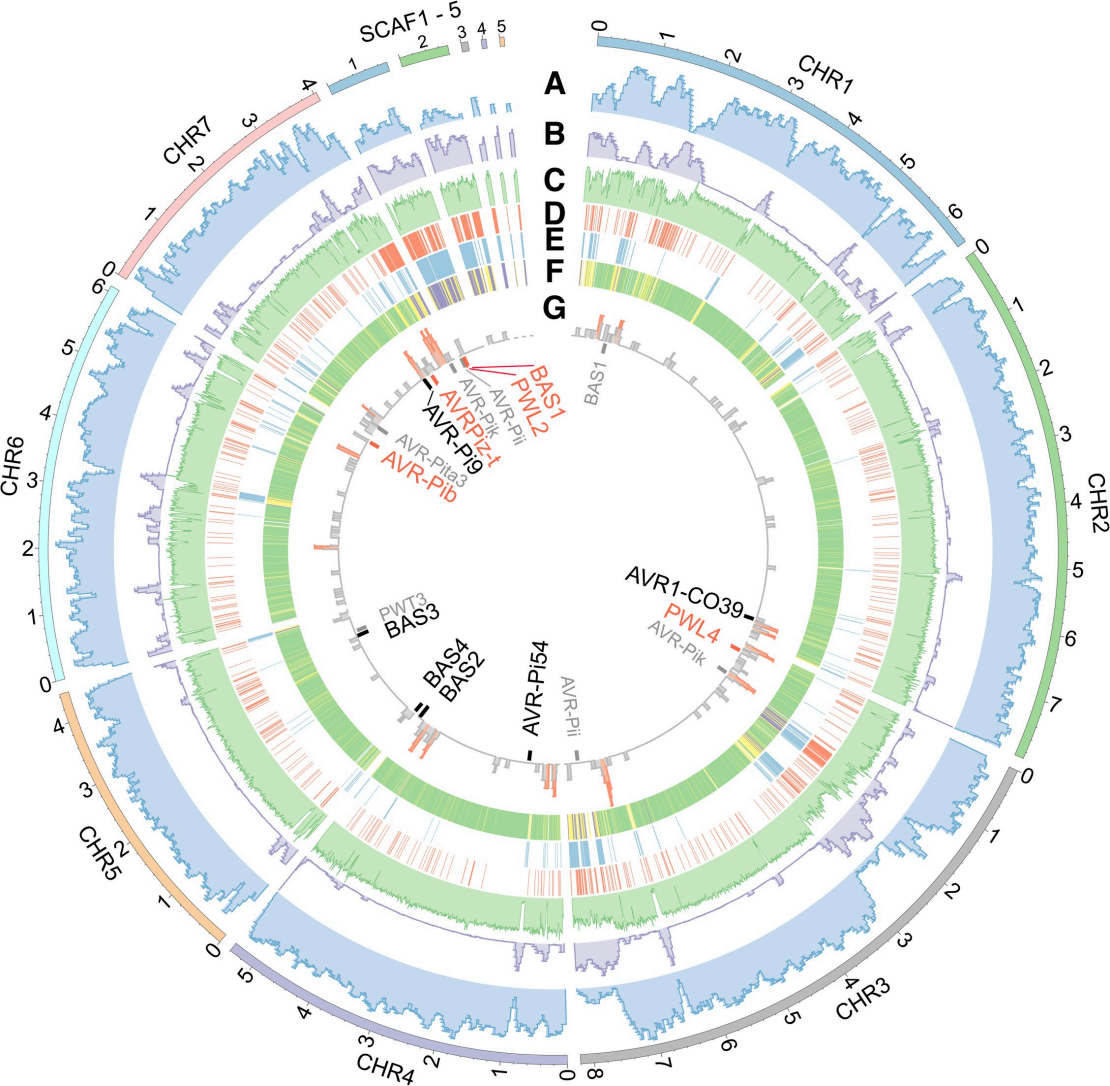
Genomic features in the B71 genome. **(A)** Density of predicted genes. **(B)** Density of repetitive sequences. **(C)** GC content. **(D,E)** Regions subjected to CNplus **(D**) or CNminus (**E**) in at least one of eight isolates (P3, B2, B51, T25, Py22.1, Py5020, P28, and P29). **(F)** CNequal regions among all isolates, CNplus or CNminus regions, and regions subjected to both changes, are colored in green, yellow, and purple, respectively. **(G)** Distribution of putative effector genes and known effector genes. The 100 kb regions with at least 3 putative or known effector genes are highlighted in red. Genes with at least 90% similarity to known effector sequences are labeled in red and black, with and without strong evidence of *in planta* specific expression from our RNA-Seq analysis, respectively. All genes of *in planta* specific expression were highly up-regulated at 40 HPI. Genes with a 50–90% similarity to published effector sequences are in gray. Note that numbers outside the outmost circle are physical coordinates in megabase pairs.

Telomere repeat sequences (TTAGGG)_n_ or *M*. *oryzae* telomeric retrotransposons (MoTeRs) that integrate in telomere repeats [28] were identified on both ends of chromosomes 2, 4, 5, 6, 7 and on one end of chromosome 1, indicating that B71Ref1 is a near end-to-end assembly. The B71Ref1 and MG8 assemblies show high end-to-end co-linearity for chromosomes 2, 4, 5, and 7 (**Fig 2C, S2 Data**). A two-megabase rearrangement was identified between chromosomes 1 and 6, of which part of chromosome 1 of MG8 was located on chromosome 6 of B71. The rearrangement was supported by eight pairs of LIEP sequences (**S3 Fig**) and by 50 single PacBio long reads. This rearrangement is not MoT specific because it was also observed in a MoO field isolate, evidenced by a long PacBio assembled sequence spanning both chromosome 1 and chromosome 6 of MG8 [29]. A large sequence in B71Ref1, from 1.3 to 2.9 Mb on chromosome 3, was absent in MG8. The unanchored 70–15 MG8 contig, supercont8.8, was mapped at the beginning of B71 chromosome 7, implying supercont8.8 is the missing end of chromosome 7 in the MG8 reference genome. None of five unanchored scaffolds of B71Ref1 can be mapped to MG8, with the requirement of, at minimum, a 10-kb match and 95% identity. Annotation of B71 identified 12,141 genes, with 1,726 harboring signal peptide domains (**Fig 3**, **S3 and S4 Data**). Of the 248 highly conserved core set of eukaryotic genes, 243 (98.0%) orthologs from the B71 annotation were identified by CEGMA, compared to 97.6% orthologs in MG8. Therefore, completeness and annotation of the B71 genome are at least comparable to that of MG8, which was produced using Sanger sequencing and multiple technologies.

Comparison of RNA-Seq data of MoT-infected wheat from the field in Bangladesh [3] and culture-grown MoT identified 335 and 153 genes that were only expressed *in planta* and in culture, respectively (SI Materials and Methods) (**S5 Data**). Secretion signal domains occurred in 173 *in planta*-specific genes, and in 18 culture-specific genes. The *in planta*-specific genes included homologs of five MoO effector genes, including *PWL2* and *PWL4* (an inappropriately expressed homolog from a weeping lovegrass pathogen that fails to block infection of *Eragrotis* spp.) [11,12], *AVR-Pib* and *AVRPiz-t* that determine rice cultivar specificity [30,31], and the cytoplasmic effector *BAS1* [32] **(Fig 3G and S5 Data)**. The remaining 168 *in planta*-specific genes were considered putative effectors **(S6 Data)**. Both known and putative effector genes tended to be located towards the ends of core chromosomes **(Fig 3G)**. We also generated RNA-Seq data from both B71 *in planta* leaf samples enriched with fungus at 40 hours post inoculation (HPI) and from B71 grown in liquid medium, which was referred to as the second RNA-Seq experiment. Differential expression analysis identified 2,891 up-regulated genes and 2,429 down-regulated genes of *in planta* B71 samples as compared to *in vitro* cultured samples. Considering genes with high fold changes in expression (at least 16x fold-change) between the two groups, we found many more highly up-regulated genes than highly down-regulated genes *in planta* (863 vs. 44). Of 174 known or putative effector genes, 110 were highly up-regulated at 40 HPI.

### Abundant copy number variation among *M*. *oryzae* isolates

**Fig 4:**
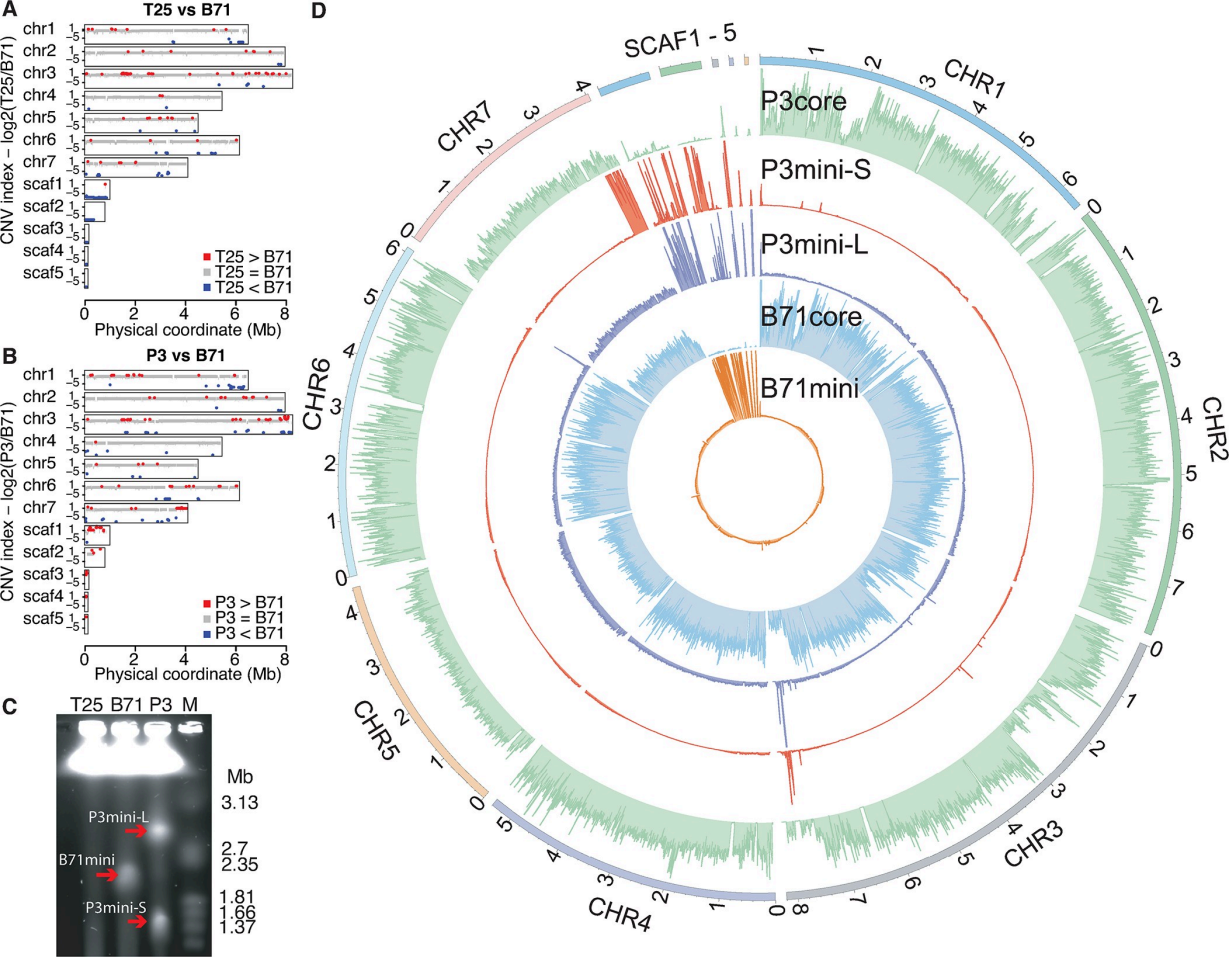
Genome comparison and mini-chromosomes. **(A,B)** Comparisons of T25 versus B71 (**A**) and P3 versus B71 (**B**). CNV index (y-axis) represents the log2 value of the ratio of read counts of a genomic segment between two isolates. Red, blue, gray lines represent CNplus, CNminus, and CNequal, respectively. **(C)** CHEF gel of three MoT isolates. Red arrows indicate mini-chromosomes. **(D)** Genome-wide distributions of read depths of 10kb bins, determined by uniquely-mapped reads, from sequencing of gel-excised core or mini-chromosomes. The 99.5% percentile of read depths per bin sets track height.

We sequenced eight additional field isolates, including less-aggressive early strain T25 isolated in Brazil in 1988 [26], five other MoT strains, a MoL strain, and a MoE strain **(S1 Fig and S1 Table)** [6]. A read depth approach was employed to detect genomic copy number variation (CNV) between B71 and each isolate, focusing on the identification of genomic regions with conserved copy number (CNequal), higher copy number (CNplus), or lower copy number (CNminus) in non-B71 isolates (**S4 Fig**). Among ~41.7 Mb of low repetitive regions, 36.4 Mb (87.3%) exhibited CNequal among all nine isolates. In total, 4.9 Mb (11.8%) displayed CNV between B71 and at least one other isolate, with 2.7 Mb (6.5%) being CNplus and 3.4 Mb (8.2%) CNminus (**Fig 3D, 3E and 3F**). Ten effector homologs [9] (*PWL4*, *AVR-Pik-chr3*, *AVR-Pi54*, *BAS1-chr1*, *BAS2*, *BAS3*, *BAS4*, *AVR1-CO39*, *AVR-Pi9*, and *AVRPiz-t*) resided in CNequal regions (chromosome identifier added to distinguish paralogs). Four (*AVR-Pii-chr3*, *AVR-Pib*, *PWL2*, and *BAS1*) were in CNminus regions and four (*PWT3*, *AVR-Pii-scaf1*, *AVR-Pib*, and *AVR-Pik*) in CNplus (**S3 Table**). CNV analysis of effector genes was supported by Illumina draft assemblies of the eight strains (**S4 Table**). Sequences from Illumina draft assemblies also showed sequence variation of some effector genes among these strains, such as DNA insertions in *PWT3* and *AVR-CO39*, two *AVR* genes governing host specificity [13,33,34]. Thus, some *AVR* homologs are equal in copy number and highly conserved across all strains, while many are subject to sequence changes, including copy number changes. Of 1.2 Mb genomic sequences exhibiting CNplus in some isolates but CNminus in others, ~819 kb (68.5%) were from the five scaffolds (scaf1-5), which constitute only 4.3% of the genome. CNV variation of sequences in the B71 scaffolds indicated they are absent in the less aggressive MoT strain T25 (**Fig 4A**). The P3 and B71 comparison, however, suggested that most scaffold sequences are duplicated in P3, an aggressive isolate from Paraguay in 2012 (**Fig 4B**). In summary, extensive copy number variation was observed among *M*. *oryzae* field isolates, especially in five scaffolds that were not anchored to the seven chromosomes.

### Dispensable mini-chromosomes of MoT strains

Variability in the five scaffolds led us to hypothesize that some or all scaffolds might correspond to mini-chromosome sequences in B71. Electrophoretic karyotypes of B71 using contour-clamped homogeneous electric field (CHEF) electrophoresis confirmed that B71, indeed, contained a mini-chromosome or multiple mini-chromosomes of ~2.0 Mb in size (**Fig 4C**). Mini- and core chromosomal DNAs were separately excised from the gel for Illumina sequencing. The five scaffolds were highly over-represented among reads obtained from the mini-chromosome DNA and highly under-represented among the core chromosome reads, confirming that all five scaffolds are from the mini-chromosome (**Fig 4D**). Roughly equal mean depths of B71 WGS reads mapped on all seven core chromosomes or the mini-chromosome supported that B71 contains a mini-chromosome. The mini-chromosome contains 192 protein-coding genes. Of those, 58.9% (113/192) of the genes were expressed (**S5 Data**). Approximately half expressed genes (N = 56) were highly regulated in expression with at least 16 fold changes comparing 40 HPI *in planta* samples with *in vitro* cultured samples, and, significantly, they were all up-regulated *in planta*, which indicated that genes in the mini-chromosome are likely to be associated with pathogenicity. Of 113 expressed genes, 23 were functionally annotated. Notably, the mini-chromosome contains four of all six genes in the genome that encode plasma membrane fusion proteins, and all four were highly up-regulated *in planta* at 40 HPI. Three functionally annotated genes exhibited *in planta* specific expression in the field samples or the B71 *in planta* leaf sheath samples, namely BSY92_12116, BSY92_11977, and BSY92_12070, encoding endochitinase B1, a gentisate 1,2-dioxygenase, and a heat-labile enterotoxin (a putative effector gene), respectively. A transcriptional regulatory gene, an *Sge1* homologous gene (BSY92_12088), governing expression of secondary metabolite biosynthetic genes [35] was highly up-regulated *in planta*. Most other functionally annotated expressed genes are associated with putative enzymatic activities. A gene BSY92_11993 encoding ubiquitin-like-specific protease 2 was expressed in both *in planta* and *in vitro* cultured samples, but it was highly up-regulated *in planta*. Gene ontology (GO) enrichment analysis identified that cysteine-type peptidase activity (GO:0008234, p-value = 0.0001) was over-represented in genes on the mini-chromosome (**S7 Data**). Eight out of all 11 genes associated with cysteine-type peptidase activity are located on the mini-chromosome, and 7 out of these 8 were expressed in either *in planta* or *in vitro* cultured samples.

**Fig 5:**
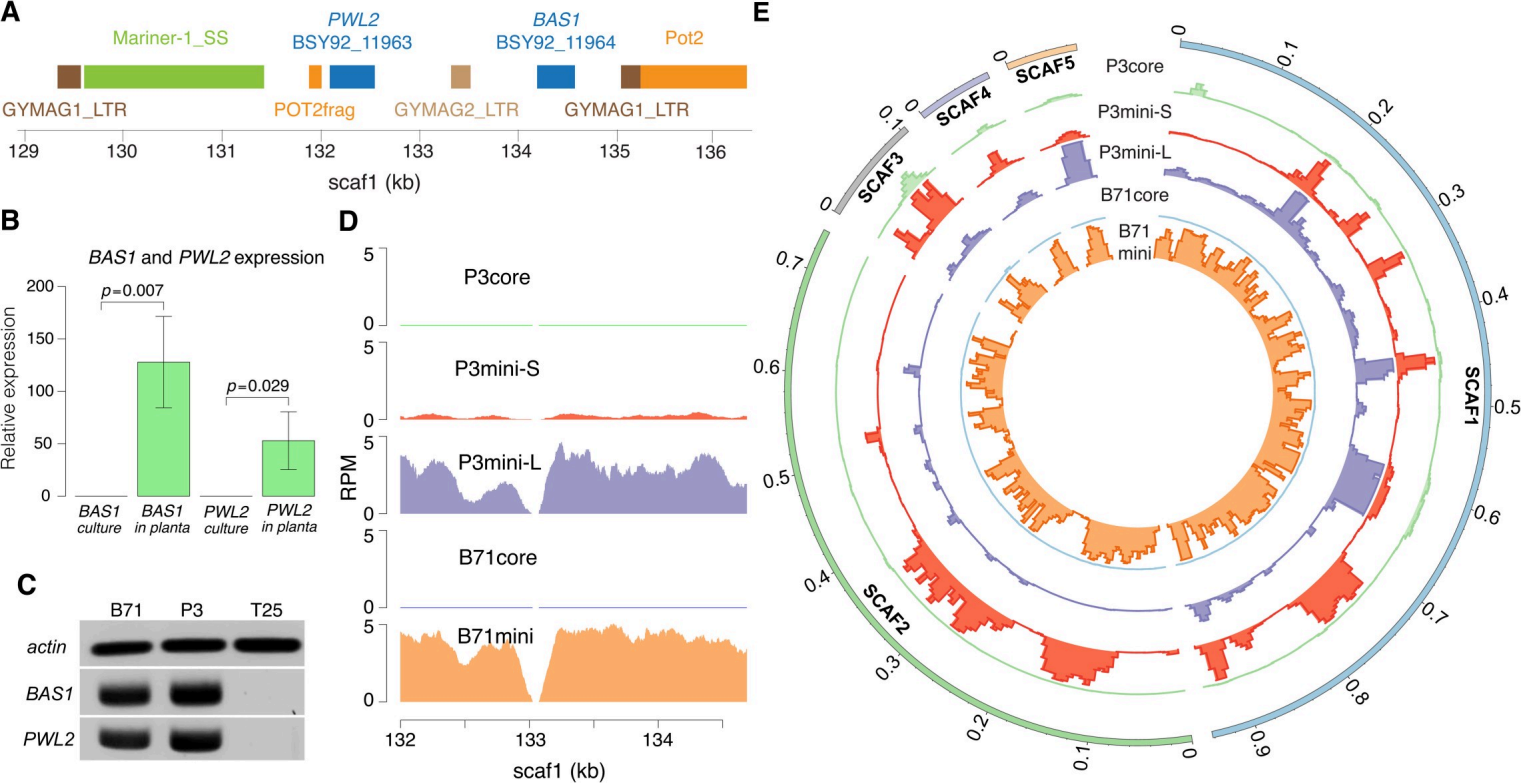
Sequences on mini-chromosomes. (**A**) Transposon sequences around *PWL2* and *BAS1* on the B71 mini-chromosome. Pot2frag is a partial Pot2 sequence. Mariner-1_SS is from a DNA transposon TcMar-Fot1 subclass. (**B**) Quantification of *BAS1* and *PWL2* gene expression via qRT-PCR. P-values are from t-tests of expression between cultured and *in planta* samples using the B71 isolate. Standard deviation is shown on each bar. (**C**) Genomic DNAs of B71, P3 and T25 were subjected to PCR with primers of each of *actin*, *BAS1*, and *PWL2* genes. (**D**) Distributions of depths of uniquely mapped reads from WGS sequencing of gel-excised core or mini-chromosomes in the *PWL2*-*BAS1* region. Due to different sizes of core and mini-chromosomes, each RPM (reads per million of total reads) was normalized by multiplying the ratio of the estimated chromosome size (e.g., the size of B71mini) to the B71 core genome size. The total chromosome sizes of B71core, B71mini, P3core, P3mini-L, and P3mini-S are 43 Mb, 2 Mb, 43 Mb, 3 Mb, and 1.5 Mb, respectively. (**E**) Distributions of read depths of 10kb bins from WGS sequencing of gel-excised core or mini-chromosomes on the B71 mini-chromosome (scaf1-5). Only uniquely mapped reads were used to determine read depths. For each track, the 99.75% percentile of read depths per bin was used to set the track height.

Known effector genes *PWL2* and *BAS1* (**S5 and S6 Figs**), which are located on different core chromosomes in MG8, were located immediately adjacent to one another and surrounded by various transposon sequences on the B71 mini-chromosome (**Fig 5A**). This configuration was supported by 211 PacBio long reads and by Sanger sequencing of a PCR product obtained with a *PWL2* and *BAS1* primer pair (**S7 Fig**). No *PWL2* or *BAS1* homologs, with at least 70% identity, were identified on core chromosomes, supported by an under-represented sequencing coverage on the *PWL2* or *BAS1* regions from CHEF sequencing of B71 core chromosomes (**Fig 5D**). Both genes exhibited *in planta-*specific expression on the mini-chromosome (**Fig 5B and S8 Fig**). Therefore, mini-chromosomes harbor effector genes that show similar *in planta*-specific expression patterns to effector genes residing on core chromosomes.

Further CHEF analyses showed no evidence of mini-chromosomes in T25 and supported at least two mini-chromosomes in P3, consistent with predictions from the CNV results. The P3 mini-chromosomes are ~1.5 Mb and ~3 Mb in length (**Fig 4C**). Sequences of both P3 mini-chromosomes exhibited similarities to the B71 mini-chromosome but also marked differences (**Fig 4D and Fig 5E**). The large P3 mini-chromosome contained both *PWL2* and *BAS1* genes (**Fig 5C and 5D**), plus it harbored ~33 kb (assembly location 6,007 to 6,039 kb) of duplicated DNA from a region near the end of chromosome 6. This duplicated DNA segment included a homolog of the MoO effector *AVR-Pib* [30]. In contrast, the small P3 mini-chromosome lacked the *PWL2* and *BAS1* genes, but it contained a duplication of approximately 0.39 Mb of the chromosome 7 end (assembly location ~3.65 to 4.04 Mb) (**Fig 5D**). Retention of this segment in the core chromosome explains the large CNplus segment at this region of P3 chromosome 7 (**Fig 4B**). The CNV result indicated that both sequences of ends of chromosome 6 and chromosome 7 found in separated mini-chromosomes have only one extra copy, supporting that P3 mostly likely has no more than two mini-chromosomes. Notably, this segment contained five putative effector genes and a homolog of the known MoO effector gene *AVR-Pik* [36]. Another notable region from the end of chromosome 3 was present in both P3 mini-chromosomes, but not present in the B71 mini-chromosome (**Fig 4D**). Sequencing P3 core chromosomes identified sequences homologous to the B71 mini-chromosome that were not present in B71 core chromosomes (**Fig 4D**). Taken together, these three MoT mini-chromosomes contain different sets of known or predicted effector genes and other core-chromosome end sequences, which are either missing or duplicated on the core chromosomes of the same or other strains. The highly variable structure of MoT mini-chromosomes indicates frequent acquisition of sequences from core chromosomal ends.

### Repetitive sequences in B71 core- and mini-chromosomes

**Fig 6:**
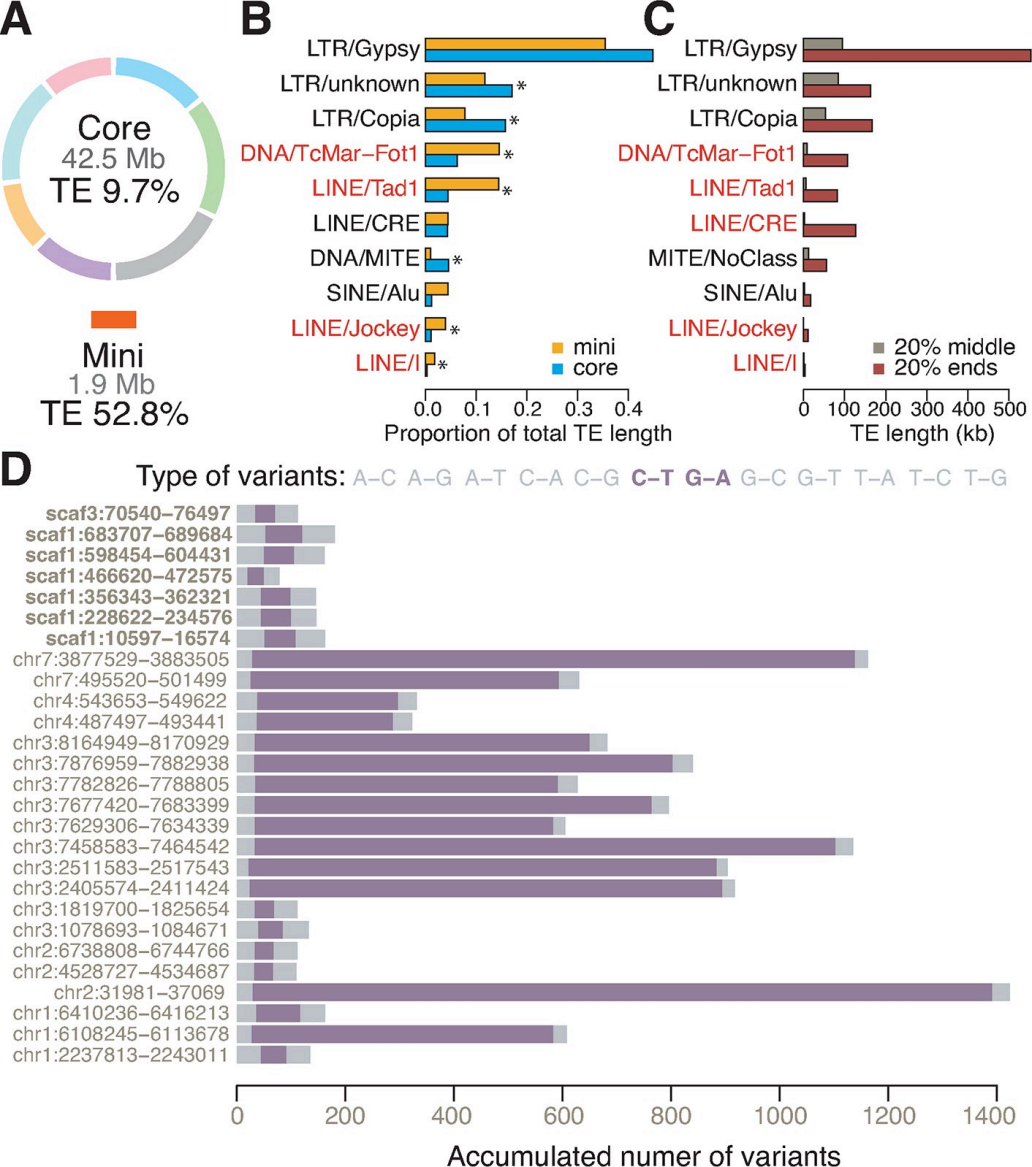
Comparison of repeats between core and mini-chromosomes. **(A)** Proportions of transposable elements (TE) in core and mini-chromosomes. **(B)** Barplots of proportions of each subclass out of total transposon sequences in core and mini-chromosomes. Subclasses with significantly proportional differences between core and mini-chromosomes are labeled with an asterisk (*), and subclasses over-represented in mini-chromosomes are highlighted in red. **(C)** Barplots indicate length of transposon subclasses in 20% ends and 20% middle regions of core chromosomes. Subclasses with at least 10-fold reduction in 20% middle versus 20% ends are highlighted in red. **(D)** Distribution of different variant types in MGR583 homologs relative to a reference MGR583 (e.g., A-C represents base A in reference MGR583 has changed to C in its homologs). RIP-type mutations are highlighted in purple. Name labels show the genomic coordinate of each homolog.

Repeat annotation showed approximately 12.9% of the B71 genome consisted of transposons and other repetitive elements, and transposons accounted for 9.7% and 52.8% of the core and mini-chromosomes, respectively (**Fig 6A and S5 Table**). Many of the transposons that were over-represented in the mini-chromosome occurred frequently on chromosome arms, particularly at chromosome ends (**S9 Fig**). Four transposon subclasses made up a greater proportion of the total transposon sequences on the mini-chromosome versus core chromosomes, including three LINEs (Tad1, Jockey and I) and the DNA transposon TcMar-Fot1 (**Fig 6B**). These four are among the top five elements enriched in the core chromosomal 20% ends relative to the 20% middle core chromosome regions (**Fig 6C**). Besides similarities in transposon composition between chromosome ends and the mini-chromosome, alignment of the B71 mini-chromosome sequence to core chromosomes identified duplications of >10 kb fragments with at least 95% identity. Duplications were located at ends of chromosomes 3, 4, and 7 (**S9 Fig**), and they were highly enriched for telomere-associated MoTeRs (LINE/CRE element). Therefore, a subset of MoT transposons is implicated in dynamic interactions between MoT mini-chromosomes and core chromosome ends.

Nucleotide composition analysis indicated that, overall, repetitive sequences along core chromosomes were highly negatively correlated with GC content (**Fig 3B and 3C**, **S10 Fig**). However, the highly negative correlation did not hold in the mini-chromosome, which is highly repetitive while maintaining relatively high GC content (**S10 Fig**). Repetitive sequences in many fungi, including *M*. *oryzae* MoT strains, are subject to repeat-induced point (RIP) mutation resulting in C-to-T or G-to-A transitions and, thereby, leading to reduced GC content [37–40]. Given higher GC content of repetitive sequences in the mini-chromosome versus core chromosomes, we explored the possibility of different levels of RIP in these genomic regions by assessing their RIP-type mutation rates. Of six high-abundance transposons examined, all exhibited reduced levels of RIP-type mutations in the mini-chromosome relative to core chromosomes (**S11 Fig**). We examined transposons MGR583 (LINE/Tad1 element) and Pot2 (DNA/TcMar-Fot1 element) that are present with multiple copies in both core and mini-chromosomes. RIP analysis indicated that no sequences of MGR583 (N = 7) or Pot2 (N = 22) from the mini-chromosome were subjected to extensive RIP-type mutations, while 14/20 MGR583 and 3/19 Pot2 from core chromosomes contained abundant RIP-type mutations (**Fig 6D and S12 Fig**). Therefore, unlike transposons in core chromosomes, transposons in MoT mini-chromosomes do not appear to be inactivated by the RIP genome defense mechanism.

## Discussion

The B71 reference genome for the wheat blast fungus has shown a high degree of macrosynteny for the core chromosomes relative to the rice pathogen reference genome 70–15 (MG8), which supports the recent report maintaining *M*. *oryzae* as a single species [6]. In contrast, mini-chromosomes present in B71 and another recent MoT field isolate P3 (P3-large and P3-small mini-chromosomes) are highly variable, with each one containing shared and different MoO effector homologs, putative effector genes, and other sequences from core chromosome ends. The B71 and P3-large mini-chromosomes contain the only copies of known MoO effectors *PWL2* and *BAS1* in these strains and neither gene was present in the early MoT strain T25, which we show lacks mini-chromosomes. *PWL2* and *BAS1* are located on different core chromosomes in 70–15, but they are found side-by-side on the B71 mini-chromosome. Both effectors show similar *in planta* specific expression on the MoT mini-chromosomes and on the MoO core chromosomes. Only the P3-large mini-chromosome contains a homolog of the MoO *AVR-Pib* gene, and only the P3-small mini-chromosome contains a homolog of *AVR-Pik*. Each mini-chromosome contains many other sequences that are either duplicated from core chromosome ends or missing from core chromosomes altogether. In one case, a P3 core chromosome sequence was homologous to the B71 mini-chromosome but not present in B71 core chromosomes. Taken together, our findings provide new insight on the *M*. *oryzae* two-speed genome [15] previously known to involve effector localization in transposon-rich regions near chromosome ends. We expand understanding of this effector compartment to include two apparently interchangeable regions, non-dispensable core chromosome ends coupled to dispensable mini-chromosomes.

We show that the *M*. *oryzae* accessory mini-chromosomes have a unique set of properties relative to accessory chromosomes in other fungi, including the well-studied accessory chromosomes in *Fusarium* species [41,42] and in *Zymoseptoria tritici* (syn. *Mycosphaerella graminicola*) [43,44]. *M*. *oryzae* mini-chromosomes, like lineage-specific chromosomes in *Fusarium* spp. [41,42] and the mini-chromosome in *Leptosphaeria maculans* [45] contain multiple genes associated with virulence and host-specificity. However, extensive recombination with core chromosomes has been so far only observed in *M*. *oryzae* mini-chromosomes. The rich set of accessory chromosomes in *Z*. *tritici* lack genes with an obvious role in pathogenicity, although some contribute quantitative pathogenicity effects in some strains [46]. The *Z*. *tritici* accessory chromosomes appear to be relatively ancient based on apparent survival through at least one speciation event [43,46]. *M*. *oryzae* mini-chromosomes resemble the accessory chromosomes of *F*. *poae* in lacking signs of the fungal specific genome defense mechanism known as RIP [47], therefore differing from the mini-chromosome and AT-isochore regions of *L*. *maculans* for which RIP appears to be a major mechanism for effector gene mutation during response to *R* gene deployment [48,49]. The gene and transposable element crosstalk between the core and supernumerary genomes reported in *F*. *poae* does not preferentially involve effectors and core chromosomes ends such as we report for *M*. *oryzae* [47]. Although supernumerary chromosomes in many systems appear heterochromatic, with low levels of gene expression [21], effector genes in *M*. *oryzae* mini-chromosomes show *in planta* specific expression characteristic of these genes on core chromosomes. Therefore, mini-chromosomes in the wheat blast pathogen differ in degree of variability of effector gene content and extent of recombination with core chromosome ends compared to dispensable chromosomes characterized so far in other fungi.

The mechanism for sequence exchange between core- and mini-chromosomes is unknown. However, the enrichment in mini-chromosomes of multiple subclasses of LINE retro-transposons and a DNA transposon that are also enriched at core chromosome ends, points to a transposon-mediated recombination mechanism involving non-allelic homology. Such a mechanism has been shown to facilitate genome rearrangements in another phytopathogenic fungus [50]. In contrast to seemingly RIPed core chromosome copies, the multiple copies of both MGR583 (LINE element) and Pot2 (DNA element) in the mini-chromosomes are nearly devoid of RIP-type mutations. This suggests that transposons on the mini-chromosomes remain active, facilitating multiplication and recombination. Telomere-associated MoTeR elements, found in MoL strains but not in MoO strains, are present on MoT mini-chromosomes. MoTeR elements have been reported to account for the extreme sequence variability of MoL telomeres compared to MoO telomeres [28], suggesting these elements might enhance mini-chromosome dynamics in MoT and MoL strains through destabilization of telomere regions. Transposon-rich genomic regions have been linked to increased sequence and structural variation in fungal plant pathogens [15,25,46]. Therefore, transposon-rich mini-chromosomes that also carry a number of genes, including many putative effectors, likely serve as genomic hotspots promoting genomic variation. Exceptional genomic variation produced in mini-chromosomes, and capable of flowing into core chromosomes, could accelerate the evolutionary potential of the pathogen.

Dynamic interchange between mini-chromosomes and core chromosome ends would contribute to *AVR-Pita1* effector gene mobility, which is especially characteristic of rice pathogens [18]. *M*. *oryzae* rice pathogens are notorious for their ability to rapidly overcome deployed *R* genes. *AVR-Pita1 and AVR-Pita2*, which each confer avirulence to rice carrying the corresponding *Pita* resistance gene, belong to a subtelomeric gene family (**S4 Table**) and show a high rate of spontaneous mutations, including frequent deletions [51,52]. *AVR-Pita1 and AVR-Pita2* occur in zero, one or more copies in different *M*. *oryzae* isolates and show highly variable genomic locations, usually near ends on core chromosomes 1, 3, 5, 6, 7; in 3 separate locations on chromosome 4; and on supernumerary chromosomes [18]. In contrast, *avr-pita3*, which lacks AVR activity, is stably located on chromosome 7 across the host-adapted lineages of *M*. *oryzae*. Therefore, extremely high genomic mobility, particularly of *AVR-Pita1*, appears to be a response to the periodic deployment of the *Pita* gene in rice. Mini-chromosomes would provide a population-wide repository for *AVR* genes that are deleted from individual strains and a means for rapid loss of *AVR* gene function from individual strains, because mini-chromosomes are frequently lost during meiosis and mitosis [22–24,53]. Individual strains lacking *AVR-Pita1* could regain it through acquiring *AVR-Pita1* containing mini-chromosomes from other individuals through the parasexual cycle and lateral gene transfer [18]. This would explain how the gene became integrated into new locations on the core chromosomes, typically at chromosome ends. The dynamic coupling we report between mini-chromosomes and core chromosome ends supports the multiple translocation hypothesis for *AVR* genes responding to periodic negative selection pressure of *R* gene deployment. Collectively, we propose that the mini-chromosome plays a role for gene movements like a shuttle, in which mutation, duplication, loss, and rearrangements of DNA occur at a faster pace than normal genomic changes, hence, accelerating genomic evolution for adaptation.

Growing evidence suggests that avirulence-conferring *PWL* family members (**S4 Table)** may be undergoing multiple translocation similarly to *AVR-Pita* family members [18]. *PWL2* from a rice isolate and *PWL1* from an *Eleusine* isolate each confer avirulence toward *Eragrostis* spp. [11,12]. The well-studied *PWL2* gene, like *AVR-Pita1*, occurs in zero to four copies in different strains and is subject to frequent spontaneous deletion [12]. Genetic analyses showed that *PWL2* and *PWL1* map to different chromosomal locations, with *PWL1* linked to a telomere. Homology between the *PWL2* and *PWL1* genes begins 70 bp upstream of the *PWL1* initiation codon and ends immediately after the stop codon, and sequences beyond this conserved region are completely unrelated. In contrast, the apparently allelic, non-AVR conferring *PWL3* and *PWL4* genes mapped to a third genomic region and share conserved flanking sequences [11]. Two copies of *PWL2* are present on chromosomes 3 and 6 in the reference rice genome MG8, the intact *PWL2* sequence was found in three assembled contigs of a highly aggressive rice isolate 98–06 [54], and we report that *PWL2* resides on a mini-chromosome in some wheat pathogens. Further research is needed to track chromosomal dynamics of *PWL2*, as well as *PWL1*, in host-adapted forms of *M*. *oryzae*. *AVR-Pita1* effector gene mobility is reported to be in response to periodic deployment of the corresponding *Pita* gene in rice, raising the question of comparable selection pressure that might be acting in the *Eragrostis* system. Introduction of weeping lovegrass, native to South Africa, and other *Eragrostis* spp., around the world for forage and erosion control in the past decades could have provided conditions promoting loss and recovery of *PWL* family members.

Our results will inspire further exploration of function and evolutionary roles of mini-chromosomes in the fungal phytopathosystem, and facilitate answering important questions for blast on wheat and other cereal crops. Our early MoT strain T25, isolated in 1988, lacks mini-chromosomes, as was previously reported for 7 other MoT strains isolated in Brazil between 1986 and 1988 [23]. This raises the question of whether mini-chromosomes have contributed in any way to the enhanced aggressiveness characteristic of recent field isolates such as B71 and P3. It is critical to monitor further evolution, including potential recombination with other *M*. *oryzae* pathotypes, of the complex MoT population in South America and the initially clonal MoT population in South Asia [1,4]. Localization of the *PWL2* host species-specificity gene on mini-chromosomes in wheat pathogens raises the question of a role for mini-chromosomes in host jumps. Effector gene dynamics, so far only associated with a small number of MoO *AVR* effector genes corresponding to periodically deployed *R* genes, raises the question of what roles known MoO *AVR* effector homologs and *BAS1* (lacking known *AVR* activity in MoO strains) play in wheat infection by MoT strains. Finally, it is critical to identify and deploy effective wheat blast resistance.

## Materials and methods

Detailed description of materials and methods is included in **S1 Text**.

### Genetic materials

All *M*. *oryzae* strains examined were field strains from South America (**S1 Table**). MoT isolates B71, T25, and P3 were isolated in Bolivia (2012), Brazil (1988), and Paraguay (2012), respectively. All work with living wheat blast fungus in the U.S. was performed with proper USDA-APHIS permits and monitoring in BSL-3 laboratories in the Biosecurity Research Institute at Kansas State University.

### DNA extraction

Single spore isolates of each pathogen strain were cultured in complete medium for mycelium propagation. Mycelium was harvested and frozen using liquid nitrogen. To avoid excessive mitochondrial DNA, mycelial nuclei were collected by gradient centrifugation as described [55]. The CTAB (cetyltrimethylammonium bromide) DNA extraction method was applied to isolate genomic DNA from the nuclear samples [56].

### B71 genome sequencing and assembly

The 3–20 kb WGS libraries were constructed using B71 nuclear genomic DNAs. The library was sequenced with P6-C4 chemistry on ten SMRTcells of PacBio RS II. Nuclear genomic DNAs were also subjected to 2x250 bp paired-end Illumina sequencing. To increase the assembly continuity, LIEP was devised and used to generate 20–30 kb long-distance paired sequences for scaffolding. PacBio long reads were assembled using the Canu pipeline [57]. Self-correction using PacBio reads did not correct all PacBio sequencing errors. Illumina reads and the Illumina assembly sequences assembled using DISCOVAR de novo [58] were both utilized for further error correction. The resulting assembled contigs were scaffolded using LIEP long-distance paired sequences with the software SSPACE [59].

### The first and second RNA-Seq experiments

Two RNA-Seq experiments were performed. In the first RNA-Seq experiment, an *in vitro* cultured mycelium sample was collected for the total RNA extraction using RNeasy Plant Mini Kit. Total RNA was used for RNA sequencing on a MiSeq to generate 2x150bp paired-end data. Clean data after adaptor and quality trimming were *de novo* assembled using Trinity [60], which were then aided in genome annotation.

In the second RNA-Seq experiment, we attempted to compare B71 gene expression *in planta* and *in vitro* culture with three biological replicates in each group. RNAs of *in planta* samples were isolated from B71-infected epidermal cells of leaf sheaths from 3–4 weeks old wheat plants at 40 HPI. The B71 *in vitro* culture RNAs were extracted from mycelium grown in liquid swirling cultures with minor modifications to the method of Mosquera et al. [32]. The total RNAs were subjected to library preparation for mRNA sequencing to produce single-end 75bp reads. Clean data after adaptor and quality trimming were aligned to the B71Ref1 reference genome with STAR [61]. Read counts per genes were used for differential expression analysis with DESeq2 [62] with 1% false discovery rate (FDR) as the threshold to declare significantly differentially expressed genes between *in planta* and *in vitro* culture groups [63].

### Genome annotation

A Maker pipeline was used for the B71 genome annotation [64]. Both evidence-driven prediction and *ab initio* gene prediction were employed [65]. Transcriptional evidence was provided using assembled sequences from RNA sequencing data of the B71 strain that was cultured in media and field wheat leaf samples infected by Bangladesh wheat blast strains, which were genetically almost identical to B71. CEGMA was used to assess the completeness of the genome assembly or annotation [66].

### Identification of expressed genes and putative effectors

Publically available RNA-Seq data of MoT infected wheat were used as *in planta* expression data to compare with *in vitro* culture RNA-Seq data from the first RNA-Seq experiment. Field RNA-Seq data includes samples 5 and 7 from Bangladesh wheat fields [3]. These MoT isolates have been demonstrated to be almost identical to B71. All data from samples 5 and 7 were merged to represent field *in planta* transcriptomes. Genes with read abundance higher than 0.1 FPKM (fragment per kilobase of coding sequence per million reads) in either *in planta* or in culture samples were considered to be expressed genes. Genes with read abundance higher than 1 FPKM from the *in planta* data set but no reads from the cultured sample were considered to be *in planta* specific expression. *In planta* specific genes containing classical signal peptide domains [67] were considered putative effectors.

### Analysis of copy number variation between strains

Read depth approach was employed to identify CNV between each of some *M*. *oryzae* strains and B71 for each of sequence bins (e.g, 300 bp). Segmentation with the R package of DNACopy was performed to identify genomic CNV segments merged from multiple bins [68].

### CHEF karyotypes of MoT strains and mini-chromosome sequencing

MoT protoplasts were prepared and mixed with 1.5% low melting-temperature agarose [23]. Suspensions were loaded into disposable plug molds. Protoplasts in plugs were lysed with proteinase K and washed. A Biorad CHEF electrophoresis system was used for separating chromosomes embedded in the plugs. After the CHEF gel electrophoresis, DNAs from individual mini-chromosomes, and from core chromosomes as one unit, were excised and purified from the agarose gels. Purified DNAs were subjected to Illumina 2x151 bp paired-end sequencing.

### Analyses of repetitive sequences

Repetitive sequences were identified using MGEScan [69], LTR_Finder [70], LTRharvest [71,72], and RepeatModeler (github.com/rmhubley/RepeatModeler). Merging discovered repetitive sequences and previously characterized *M*. *oryzae* repeats [73] produced a non-redundant database, which served as a repeat library to identify repeats in the B71 genome using RepeatMasker (www.repeatmasker.org). Some transposable elements were subjected to analysis of RIP-type polymorphisms, nucleotide changes of C to T or G to A.

## Supporting information

S1 Text Supplementary information. (DOCX)

S1 Fig Phylogenetic tree of *M*. *oryzae* strains showing the major crop-specific lineages, also known as pathotypes. These are: *Oryza* pathotype (MoO, 44 Strains); *Setaria* pathotype (MoS, 4 strains); *Eleusine* pathotype (MoE, 6 strains); *Triticum* pathotype (MoT, 21 strains); and *Lolium* pathotype (MoL, 16 strains). Strain branches in each of five pathotypes were labeled with the same color as the pathotype identifier. Assembly data of each strain were utilized to identify polymorphisms and construct the phylogeny with the neighbor-joining tree estimation. Strains selected in this study are highlighted with red dots. Host species on which each strain was isolated from the field are indicated (e.g., T) by: B, *Brachiaria*; Br, *Bromus*; E, *Eleusine*; Er, *Eragrostis*; F, *Festuca*; L, *Lolium*; O, *Oryza*; S, *Setaria*; St, *Stenotaphrum*; T, *Triticum*. The strain G 4091-5-8, which infects both *Eragrostis* spp. and *Eleusine* spp., was obtained in a laboratory cross between E G22 and Er G17. Strains Py22.1 and Py5020 are described in Pieck *et al*, 2017; and all other non-MoO strains are described in Gladieux *et al*, 2018.
(TIF)

S2 Fig Distribution of PacBio raw reads. The number of reads, the median length, the N50, the longest length, the total length of reads greater than 5 kb, and the total length of all reads were reported. The median length and the N50 are indicated with blue and orange vertical lines, respectively. During the Canu assembly, only reads with the minimum of 14,378 bp were extracted for read correction (first step of Canu assembly). When the Quiver error correction was performed, all raw reads were used.
(TIF)

S3 Fig LIEP paired sequences on the rearrangement region on chromosome 6. The region (chromosome 6, 1–2,062,779 bp) of B71Ref1 is collinear with a partial sequence of MG8 chromosome 1, and the region beyond 2,070,228 bp of B71Ref1 chromosome 6 is collinear with MG8 chromosome 6. Blue curves showed pairs of LIEP sequences spanning the junction region, from 2,062,779 bp to 2,070,228 bp. In addition, the junction region and some flanking sequences are fully covered by 50 single PacBio long reads.
(TIF)

S4 Fig Genome comparisons between each of 8 additional *M. oryzae* strains and B71. The strains being compared are MoT strains T25, P3, B2, Py22.1, Py5020 and P29; the MoL strain P28; and the MoE strain B51. Each track represents a copy number comparison of the non-B71 isolate versus B71. The value of CNV index, which represents the log2 value of the ratio of sequencing read counts in genomic segments between two isolates of the comparison, determines vertical position on the track. Red, blue, green lines represent CNplus, CNminus, and CNequal regions relative to the B71.
(TIF)

S5 Fig Alignment of *PWL2* from isolates of 70–15 and B71. PWL2-U26313 is a partial sequence of the Genbank accession U26313. The translation start site is highlighted in a red box.
(TIF)

S6 Fig Alignment of *BAS1* homologs from isolates of 70–15 and B71. Two *BAS1* homologs were aligned with the *BAS1* from 70–15. The homolog from B71 chromosome 1 (*BAS1*-chr1) has <70% identity with *BAS1* of 70–15. MG8-BAS1 is a partial sequence of the Genbank accession FJ807764.1. The translation start site is highlighted in a red box.
(TIF)

S7 Fig Rice blast effectors *PWL2* and *BAS1*, which are on different chromosomes in MoO strains, are side-by-side on the B71 mini-chromosome. Validation of the neighboring structure of *PWL2* and *BAS1* via Sanger sequencing. The PCR product using the primers Pwl2_qRT2-R4 (primer 1) and BAS1-R (primer 2) was sequenced using these two primers separately. Green lines indicate the alignment regions on the scaf1 for two sequencing reads. Detailed alignments of two sequencing reads were shown underneath each green line.
(TIF)

S8 Fig RNA-Seq read distribution on the PWL2-BAS1 region. RNA-Seq reads distribution of *PWL2* and *BAS1*. **A**) Distributions of uniquely mapped reads from the field samples (2x101bp paired end data) and *in vitro* culture samples (2x150bp paired-end data) from the first RNA-Seq experiment. **B**) Distribution of uniquely mapped 75bp reads from three biological replicates of 40 HPI *in planta* B71 samples and three biological replicates of *in vitro* cultured B71 samples from the second RNA-Seq experiment. Note that shapes of read distributions are related to read lengths that influence mapping ability of reads. RPM (reads per million of total aligned reads) represents normalized read counts.
(TIF)

S9 Fig Distribution of selected transposon subclasses across the genome. The inset in the center shows large genome duplications (>10 kb and >95% identity) within five scaffolds, as well as between the scaffolds and the chromosomes 1–7. LINE subclass Tad1 contains the previously characterized retrotransposon MGR583 and subclass CRE contains the telomere-targeted retrotransposon MoTeR. The DNA transposon subclass TcMar-Fot1 contains previously characterized Pot2.
(TIF)

S10 Fig Scatter plot of GC percentages versus proportions of repetitive sequences of 100 kb bins. Orange and blue circles represent 100-kb bins from the B71 mini-chromosome (five scaffolds) and core chromosomes, respectively. Pearson correlations between GC percentages and proportions of repetitive sequences of 100-kb non-overlap genomic bins of the mini-chromosome and core chromosomes.
(TIF)

S11 Fig Boxplots of proportions of RIP-type changes in multiple transposable elements. Genomic sequences of each transposable element were aligned to corresponding transposon sequences from the RepeatMasker database as the reference sequences. Polymorphisms were determined for each sequence that exhibits at least 60% overlap with the reference sequence. For each transposon element, a t-test was performed to test the null hypothesis that the mean proportions of RIP-type variants out of the total mismatches of transposons located in core chromosomes was not different from that of transposons located at the mini-chromosome. P-values of t-tests were shown on the top of each boxplot.
(TIF)

S12 Fig Distribution of different variant types on Pot2 homologs. Sequences of Pot2 homologs were aligned with the reference Pot2. Mismatching variants of each Pot2 homolog relative to the reference Pot2 were categorized based on nucleotide changes. All twelve variant types were listed on the top. For example, A-C represents base A on the reference Pot2 is changed to base C on Pot2 homologs. Each row shows the accumulated number of variants of a Pot2 homolog at the order of type of variation listed on the top. Two RIP-type mutations were highlighted in purple. Labels on the left show genomic locations of each Pot2 homolog.
(TIF)

S13 Fig Linker design for LIEP. Two synthetic oligos with random barcodes and Illumina compatible sequence were annealed by 21 bp overlapping sequence (green italic sequences of Lo3b and Lo4b). The annealed product was then filled to form a double-stranded linker DNA (top sequence). The design of the link was shown. N(17) and N(21) indicated 17 and 21 randomly synthesized nucleotides, respectively. The linker sequence contains other IUPAC nucleotide code (e.g., H = A, C or T).
(TIF)

S1 Table Strains used in this study. (DOCX)

S2 Table Statistics of PacBio and Illumina assemblies. (DOCX)

S3 Table CNV overlapping effectors genes. (DOCX)

S4 Table Copy number of effectors based on assembled sequences. (DOCX)

S5 Table Summary of repetitive elements of the B71 MoT genome. (DOCX)

S6 Table List of primers or oligos used in this study. (DOCX)

S1 Data Difference between PacBio drafted sequences before Illumina correction and the B71Ref1 sequences. (VCF)

S2 Data Large genomic regions with a high identity between B71 and MG8. (XLSX)

S3 Data Functional annotation of genes. (TXT)

S4 Data GTF file of genome annotation. (GTF)

S5 Data Gene expression from RNA-Seq. (TXT)

S6 Data List of putative effectors. (XLSX)

S7 Data Gene ontology of genes. (TXT)
