# Supplementary material for: Effector gene reshuffling involves dispensable mini-chromosomes in the wheat blast fungus
Source: PLoS Genet. 2019 Sep 12;15(9):e1008272. doi: 10.1371/journal.pgen.1008272 (PMC6741851; doi:10.1371/journal.pgen.1008272)
Supplement: S1 Text — (DOCX) [file pgen.1008272.s001.docx]

**S1 Text - Supplementary Information**

Effector Gene Reshuffling Involves Dispensable Mini-chromosomes in the Wheat Blast Fungus

Zhao Peng, Ely Oliveira-Garcia, Guifang Lin, Ying Hu, Melinda Dalby, Pierre Migeon, Haibao Tang, Mark Farman, David Cook, Frank F. White, Barbara Valent^*^, Sanzhen Liu^*^

^*^Correspondence to: [bvalent@ksu.edu](mailto:bvalent@ksu.edu) or [liu3zhen@ksu.edu](mailto:liu3zhen@ksu.edu)

Materials and Methods

Pathogen strains and Biosafety-Level 3 (BSL-3) research

Fungal strains used are described in **S1 Table**. MoT strain B71 was isolated from a wheat field in Bolivia in 2012. MoT isolates T25 and P3 were collected from Brazil and Paraguay in 1988 and 2012, respectively. All work with living wheat blast fungus was performed in BSL-3 laboratories in the Biosecurity Research Institute at Kansas State University, under conditions authorized by permits from the USDA Animal and Plant Health Inspection Service. All strains are permanently stored in a collection maintained in the Foreign Disease-Weed Science Research Unit at Fort Detrick in Maryland.

Agar media for fungal growth

Fungal isolates were grown on oatmeal agar plates or on rice polish agar plates. To prepare oatmeal agar plates, fifty grams of rolled oats were heated in 500 mL of water at 70˚C for 1 hr. The suspension was then filtered through four layers of cheesecloth, extracting most of the liquid. The volume of the filtrate was adjusted to 1 L with water and 15 g of agar were added before autoclaving. To prepare rice polish agar, 25 g of rice polish and 16 g of agar were added to a liter of water and mixed well. Both agar media were autoclaved for 40 min with the flasks sitting in a pan with water to reduce boiling over of the hot media. Both media were kept in a 70˚C water bath before cooling on a stir plate and pouring plates.

DNA extraction

Single spore isolation of each MoT strain was performed to ensure genetic purity before DNA isolation. The strains were cultured on oatmeal or rice polish agar plates. For preparation of mycelium from liquid culture, twenty-five 0.2-cm^2^ blocks of agar containing B71 mycelium were excised from the surface of an oatmeal agar plate and further cut to produce the smallest pieces, maximum number of growing points, possible. All pieces were placed in 100 mL of 3,3,3 medium (3 g/L of glucose, 3 g/L of casamino acids, and 3 g/L of yeast extract) in a 500-mL flask and incubated at 24˚C with continuous rotation (120 rpm) for three days. Mycelium was harvested by passing the fungal culture through a funnel covered with sterile filter papers and patted dry using paper towels. The mycelium was then frozen using liquid nitrogen and stored at -80°C. To obtain large DNA fragments and avoid excessive mitochondrial DNA, mycelial nuclei were collected by gradient centrifugation following a protocol for megabase-sized DNA [1]. The CTAB (cetyltrimethylammonium bromide) DNA extraction method was applied for nuclear samples to isolate genomic DNA [2].

PacBio sequencing

The 3-20kb whole genome shotgun libraries were constructed using nuclear genomic DNAs of the B71 strain. The library was sequenced with P6-C4 chemistry on ten SMRTcells of PacBio RS II at the Yale Center for Genomic Analysis (YCGA).

Illumina sequencing

Nuclear genomic DNAs of the B71 strain were subjected to library preparation using a TruSeq PCR-free library protocol with the insertion size of 375 bp. 2x250bp paired-end reads were generated on an Illumina HiSeq2500 at Beijing Genomics Institute.

Construction of indexed LIEP vectors

Two synthetic sequences with random barcodes and Illumina compatible sequence were annealed, end-filled and cut by *Mfe*I and *Hind*III restriction enzymes (NEB, USA) (**S13 Fig**). DNA fragments with random barcodes were referred to as linkers. Linkers with random barcodes were then inserted into the commercialized vector pEZ-BAC (Lucigen, USA) digested by *EcoR*I and *Hind*III (NEB, USA). Three millions of clones with random barcoded vectors were pooled.

Linker sequencing of LIEP barcode-indexed vectors

Plasmid DNAs of barcode-indexed vector were extracted and Illumina adaptor primers were used to amplify barcodes for sequencing. Two steps of PCR amplification using Phusion High Fidelity DNA polymerase (NEB, USA) were applied. In the 1^st^ step PCR, the Illumina compatible primer RPtag and vector unique primer L95 (**S6 Table**) was used, following the protocol: 98˚C 30 seconds, 5 cycles of (98˚C 10 seconds, 57˚C 15 seconds, 72˚C 15 seconds), and 72˚C 2 minutes. After purification of the 1^st^ step PCR product, it was directly used in the 2^nd^ step PCR by using modified Illumina primers htF501s2 and htF701s (**S6 Table**), following the same PCR protocol in first step except changing the T_m_ to 61˚C. Libraries were sequenced on a MiSeq with 2x78 cycles at the Integrated Genomic Facility at Kansas State University and on a HiSeq2000 with 2x100 cycles at the Genome Sequencing Facility at the Kansas University Medical Center. Two barcodes of a barcode pair of each vector were extracted from each sequencing read using an in-house Perl script. Barcode data were kept only when identical barcode sequences were extracted from two paired reads. Unique barcode pairs were obtained after removing redundancy.

LIEP library preparation and sequencing

The genomic DNA of B71 strain was partially digested by *Sau3A*I restriction enzyme (NEB, USA), around 20-30 kb DNA fragments were selected to ligate with *BamH*I digested barcoded vector pool, which had been dephosphorylated with rSAP (NEB, USA) to avoid self-ligation. Eight pools of BAC clones were obtained and individual pool contained 8,000-25,000 unique clones. In total, approximately 100,000 of BAC clones were obtained. The DNA of each BAC pool was extracted using regular plasmid preparation protocol. The regular Illumina library preparation protocol was modified for sequencing the BAC ends. The DNA of each BAC pool was sheared into 500-800bp fragments in Coravis ultrasonicator (Integrated Genomic Facility, Kansas State University). End repair and dA-tailing with ddATP (Sigma, USA) of the sheared DNA was performed following the NEB Next Ultra End repair/ dA-tailing Module. T-adaptor was prepared by annealing the hta1 and hta2 oligos (**S6 Table**) under the following protocol: heating at 95˚C for 5 minutes, cooling to 25˚C at the rate 1˚C every 15 seconds, and then holding at 25˚C for 30 minutes in thermal cycler. A-tailing product and the T-adapter was ligated by T4 DNA ligase (NEB, USA). Two steps of PCR amplification using Q5 DNA polymerase (NEB, USA) were then applied. In 1^st^ step PCR, primers 501Tr1 and 701Tr1 (**S6 Table**) were used, following the protocol: 98˚C 1 minute, 12 cycles of (98˚C 10 seconds, 66˚C 15 seconds, 72˚C 30 seconds), and 72˚C 2 minutes. In the 2^nd^ PCR, different indexed primers from Illumina Truseq HT primer sets (**S6 Table**) were applied for amplifying the 1^st^ PCR products, following the same PCR conditions but only 5 cycles. The resulting DNA libraries were pooled in ratios according to the size of BAC libraries, and sent for MiSeq sequencing at the Integrated Genomic Facility at Kansas State University.

LIEP data analysis

For each BAC clone, the DNA insert was flanked by two indexed barcodes that were built in a vector. The barcodes were known based on previously vector linker sequencing. That means, based on barcode information, we can figure out a pair of two insert end sequences even through two ends were sequenced separately. The LIEP data analysis is to process sequencing data to extract barcode sequence and insert sequences, and finally determine two BAC end sequences for each clone. In detail, each LIEP raw sequence (PE reads) started with a barcode followed by the sequence of a genomic DNA insert. LIEP sequencing raw sequences were first subjected to adaptor trimming (trimmomatic-0.36), without quality trimming, using TruSeq adaptor sequences, followed by extracting indexed barcode sequences. After this step, each sequence had an associated barcode, which was then used for barcode trimming to avoid barcode contamination in sequences. Quality trimming was performed at the same time. The barcode was also used to identify the paired barcode from the same vector, which was pre-determined. For example, a pair of indexed barcodes, BC-I and BC-II, was identified. BC-1 associated reads represent sequences from one end of the insert of the BAC clone, while BC-2 associated reads represent sequences from the other end of the same BAC clone. Multiple reads of each end were obtained if a BAC clone was sequenced multiple times. We then assembled BC-1 and BC-2 associated reads separately with Celera Assembler 8.0 to obtain sequences of two BAC ends [3].

PacBio genome assembly

Only PacBio reads longer than 5 kb were used for the assembly. Canu v1.3-r7616 was selected to correct reads, to trim suspicious sequences (e.g., adaptors), and to assemble corrected and cleaned reads into unitigs [4].

Illumina genome assembly

Trimmomatic (version 0.36) was used to trim adaptor sequences of TruSeq PCR free 2x250bp sequencing reads [5]. Trimmed reads after removing adaptors were subjected to error correction with the error correction module of ALLPATHS-LG (GNERRE et al. 2011). Corrected reads were *de novo* assembled using DISCOVAR de novo [6]. The final Illumina assembly contains contigs with at least 500 bp.

Genome assembly polishing and scaffolding

The tools pbalign and Quiver were used to align PacBio reads to the PacBio assembly and polishing. Self-correction in Canu and Quiver using PacBio reads corrected most sequencing errors. However, a number of sequencing errors existed in the draft PacBio assembly, particularly on homopolymeric regions. Two rounds of additional polishing were employed to improve the assembly quality. First, trimmed clean PCR free Illumina genomic DNA sequencing reads were directly aligned to the draft PacBio assembly with the *mem* module of BWA (0.7.10-r789) [7]. Alignments were filtered using the following criteria: at least 100 bp matches with 94% identity, at least 98% coverage, and at least 40 of mapping score. Alignments passing the filtering criteria were used to call small INDELs (insertions and deletions) with GATK (version 3.3), followed by a set of filtering criteria [8]. Each INDEL required at least 10 but no more than 5,000 reads supported and the non-reference variant (an alternative sequence type relative to the draft assembly) of each INDEL site accounted for at least 90% reads of the total supporting reads covering the variant site. The corrected draft assembly with this set of INDELs was obtained by using the consensus module of bcftools [9].

To reduce errors during the correction using short Illumina reads due to false alignments, long sequences of the DISCOVAR de novo assembly were utilized for the second round of correction. Assembled sequences were aligned to the first-round corrected assembly with the mem module of BWA (0.7.10-r789) [7]. Alignments were filtered using the following criteria: at least 400 bp matches with 94% identity, at least 98% coverage, and at least 40 of mapping score. Alignments passing the filtering criteria were used to call single nucleotide variants and small INDELs (insertions and deletions) with a pipeline using samtools (mpileup module) and bcftools (the module of “call”). Again the consensus module of bcftools was implemented the second round correction, resulting in a new draft assembly. Contigs of the draft assembly were scaffolded using LIEP BAC end sequences. The software SSPACE 3.0 was used for scaffolding with the parameter of “-x 0 -k 3 -g 1 bwasw end1.fq end2.fq 30000 0.8 FR” in that end1.fq and end2.fq represent sequence FASTQ files of first ends and second ends of BACs, respectively [10]. The scaffolding required at least three pairs of LIEP sequences to establish a connection between two contigs.

*De novo* RNA-Seq assembly using the first RNA-Seq data

Mycelium grown in the 3,3,3 liquid medium as described for DNA extraction was used for RNA extraction using RNeasy Plant Mini Kit (Qiagen, USA). Total RNA was used to prepare an mRNA sequencing library that was run on a MiSeq with 2x150 cycles at the Integrated Genomic Facility at Kansas State University. The software Trimmomatic (version 0.36) was used to trim adaptor sequences of RNA sequencing reads. Only paired reads both of which are at least 50 bp after trimming were retained. Trimmed clean reads were *de novo* assembled using Trinity with the default parameter [11].

Identification of putative effectors using data of *in planta* samples from Bangladesh wheat fields

RNA-Seq data of MoT infected wheat were downloaded from Sequence Reads Achive, SRA number from ERR1360178 to ERR1360193. All data were merged to a single data set that was used as *in planta* RNA-Seq data. Both *in planta* RNA-Seq data and RNA-Seq data from a cultured sample were aligned to the B71Ref1 with STAR (2.5.2a). With the annotation file, Cufflinks v2.1.1 was used to determine read abundance per gene. Genes with read abundance higher than 1 FPKM (fragment per kilobase of coding sequence per million reads) from the *in planta* data set but no reads from the cultured sample were considered to be only expressed *in planta* or *in planta* specific expression, and vice versa. Genes that were expressed specifically *in planta* and contained classical signal peptide domains were considered putative effectors.

The second RNA-seq experiment: *in planta* 40h infected samples versus *in vitro* culture samples.

B71 was cultured on oatmeal agar plates at 24˚C under continuous light inside the BSL-3 containment laboratory at Kansas State University. Wheat cultivar Bob White was grown under controlled conditions, at 24˚C under 12 hours light period. Wheat leaf sheaths were inoculated with B71 conidia as described for rice [12]. Specifically, we used 3- to 4-week-old plants. Leaf sheaths from intermediate-aged leaves were cut into 7 cm sections. Fungal spores were harvested at a concentration of 3x10^5^ spores/mL in 0.25% gelatin (type B from bovine skin; Sigma-Aldrich G-6650). Inoculum was introduced into the hollow space enclosed by the sides of the leaf sheaths above the midvein. Inoculated sheaths were supported horizontally in a Petri dish containing wet filter paper such that the spores settled on the midvein regions. After 40 hours post inoculation (HPI), sheaths were hand-trimmed to remove the sides and expose the epidermal layer above the midvein. Lower midvein cells were then removed to produce sections three to four cell layers thick. Each sheath segment was lightly cleaned using a wet sterile swab to remove spores and mycelium on the sheath surface. The infection density and synchrony of biotrophic infection structure formation were accessed by light microscopy and heavily infected sheaths were immediately frozen in liquid nitrogen. Sheath pieces were processed one at a time to minimize time between trimming and freezing. The *in vitro* cultured mycelial samples were prepared as described above for DNA preparation. Briefly, mycelial pieces from an oatmeal agar plate were grown in 100 mL of 3,3,3 medium in a 500-mL flask at 24˚C with continuous rotation (120 rpm) for 3 days, collected, dried, and stored at -80˚C for RNA extraction. Total RNAs from mycelium and infected wheat tissues were extracted from 100 mg samples using a mirVana total RNA isolation method (Life Technologies/Thermo Fisher Scientific) according the manufacture’s protocol.

In total, three biological replicates of *in planta* RNA samples and three biological replicates of *in vitro* cultured RNA samples were prepared. Each total RNA sample was used to prepare the sequencing library using TruSeq stranded mRNA library prep kit from Illumina with insert sizes around 158bp, followed by 75 bp single-end sequencing in an Illumina NextSeq 500 the NSQ 500/550 Hi Output kit v.2.5 at the Integrative Genome Facility at the Kansas State University. Reads were subjected to adaptor and quality trimming with Trimmomatic and then aligned to the B71Ref1 genome with STAR (2.5.2a_modified) that also determined read counts per gene [13].

Differential expression (DE) analysis between 40h *in planta* samples and *in vitro* culture samples

DESeq2 (1.14.1) was used to perform DE analysis for the genes with at least 3 reads on average across all six samples [14]. The 1% false discovery rate (FDR) was used to account for multiple statistical tests [15].

Genome annotation

A Maker pipeline was used for the B71 genome annotation [16]. Both evidence-driven prediction and *ab initio* gene prediction were employed [17]. The B71 genome was repeat masked using a species-specific repeat database, which was generated by using RepeatModeler (1.0.9) (repeatmasker.org/RepeatModeler). EST evidence was provided by using Trinity assembled sequences from RNA sequencing data of the B71 strain that was cultured in media and field wheat leaves inoculated by Bangladesh wheat blast strains, which were genetically close to B71. Protein sequences of *M. oryzae* 70-15 (MG8) were provided as protein homologs. Augustus, GeneMark, and Snap were used for *ab initio* gene prediction. Snap was trained by using predicted gene models from a prior Maker run without using Snap. InterProScan (5.30-69.0) was used to perform functional analysis using default parameters [18].

CEGMA analysis

CEGMA (v2.5) was used to compare predicted genes with 248 Core Eukaryotic Genes (CEG) to assess the completeness of the genome or annotations [19].

Signal peptide analysis

Signalp-4.1 was used predicted the presence of signal peptide cleavage sites and transmembrane segments in protein sequences of each gene [20].

Gene ontology (GO) analysis

GO enrichment analysis was performed to find GO terms over-represented in the genes on the mini-chromosome. The resampling method with 10,000 samplings was employed [21].

Genome-wide variant discovery, genotyping, and construction of phylogeny

Assemblies of Mo isolates were downloaded from GenBank or from previous studies were used for the discovery of polymorphisms between each strain and the B71 reference genome. First, assembly contigs were split into multiple sequences if a contig is longer than 10 kb. Second, all resulting sequences from a strain were aligned to the B71 reference genome with BWA (0.7.10-r789). Each alignment required at least a 100 bp, at least 96% identity, at most 4% tail that can not be aligned to the reference at the ends of sequences, and at least 40 for the mapping score. GATK (version 3.3) used alignments to discover SNPs and genotyped each strain. Only bi-allelic SNP sites with at least 10% minor allele frequency, 20-500 supported sequences were retained at genotyping data. Genotyping data at SNP sites were used to construct a phylogenetic tree using an R package APE [22].

Analysis of genomic structural variation

A read depth approach was employed to examine genomic structural variation, specifically copy number variation (CNV), among some MoT strains. First, genomic bins each of which contains a certain length of non-repetitive sequences were defined. In detail, single-copy 25-mers were extracted from the B71 reference genome with Jellyfish [23]. These k-mers were mapped back to the reference genome through BWA mapping and identified uniquely mapped k-mers (unique single-copy k-mers). The genome was then scanned to find non-overlapping bins with 200 unique single-copy k-mers. Bin sizes were ranked from smallest to largest and the top 1% largest bins were removed for further analysis.

Second, Illumina reads of each strain were aligned to the B71 reference genome and read counts were determined for every defined genomic bin. Read counts were normalized by using total aligned reads. For each bin, the log2 ratio of normalized read counts was calculated between the non-B71 strain and the B71 strain (log2(non-B71/B71)), which was then used for the segmentation to identify a continuous genomic segments exhibiting similar values of log2(non-B71/B71) of bins with the R package of DNACopy [24]. The module smooth.CNA with the parameters of (smooth.region = 10, outlier.SD.scale = 4, smooth.SD.scale = 2, trim = 0.01) and the module of segment with the parameter of (alpha=0.005, nperm=10000, p.method="perm", eta=0.005, min.width=3, undo.splits = "sdundo", undo.SD = 3) were employed for the segmentation.

For a segment, the mean of log2(non-B71/B71) is close to zero if sequences of two strains are identical and no CNVs. The derivation of the mean of log2(non-B71/B71) from zero is due to sequence polymorphisms and copy number variation (CNV), including presence and absence variation (PAV). Thus, means of log2(non-B71/B71) of segments were used for the classification of segments to different categories. The classification also utilized information of read alignment coverage of segments determined by using Bedtools (the coverage module). For each segment, an alignment coverage ratio (relative coverage) was calculated by dividing the coverage of reads from a non-B71 strain to that from the B71 stain. A segment was categorized to CNVplus if the relative coverage is greater than 0.85 and the mean of log2(non-B71/B71) is greater than 0.6; segment was categorized to CNVplus segments if the relative coverage is greater than 0.85 and the mean of log2(non-B71/B71) is greater than 0.6; a segment was categorized to conserved segments, CNequal, if the relative coverage is greater than 0.85 and the mean of log2(non-B71/B71) is between -1.5 and 0.5; a segment was categorized to polymorphic segments if the relative coverage is between 0.2 and 0.8, and the mean of log2(non-B71/B71) is between -5 and -2; a segment was categorized to CNVminus segments if the relative coverage is less than 0.2, and the mean of log2(non-B71/B71) is less than -5. The mean of log2(non-B71/B71) of a segment was referred to as a CNV index of the segment.

Electrophoretic karyotypes of MoT strains

Protoplasts of MoT strains were prepared following the protocol described by Orbach et al. with some modification [25]. After resuspending fungal mycelia in 1M sorbitol (Sigma, USA), lysing enzyme (Sigma L-1412), instead of Novozym 234, was used to generate protoplasts. Purified protoplasts were resuspended in SE buffer (1M sorbitol, 50mM EDTA, pH=8.0) at a density of 3×10^9^/ml. The protoplast solution was then mixed with 1.5% low melting-temperature agarose at a ratio of 1:1 volume. The mixture was loaded into disposable plug molds (Bio-Rad, USA) and solidified for 30 minutes on ice. Protoplasts in the plugs were then lysed with proteinase K (DNAse free, Promega, USA) in the NDS buffer in a shaking water bath at 50˚C overnight [26]. The plugs were washed with 50mM EDTA (pH=8.0) buffer at least three times at room temperature and then stored at 4˚C. CHEF electrophoresis system (Bio-Rad, USA) was applied for separating chromosomal DNAs embedded in the plugs. The gels were made with 0.7% Certified Megabase agarose (Bio-Rad, USA) and run in 0.5×TBE in the cold room under electrophoresis conditions as described [27].

Sequencing of mini-chromosomes of MoT strains

After the CHEF gel electrophoresis, agarose containing B71 and P3 mini-chromosome DNAs was excised and transferred to D-tubes (Midi size) for electroelution following the provided manual (Millipore, Sigma, USA). The DNA was then concentrated by vacuum evaporation and dialyzed with distilled water on the Nitrocellulose membrane (Millipore, Sigma, USA) to remove salt. Meanwhile, B71 and P3 core chromosomes were excised as well and purified by Qiagen gel purification kit. Illumina TruSeq libraries were constructed with the purified DNA. Libraries were sequenced with 2x151 bp on a MiSeq in Integrated Genomic Facility at Kansas State University.

Genomic PCR amplification of *PWL2* and *BAS1*

Using the genomic DNA of strains B71, P3 and T25 as templates, genes were amplified by Q5 high-fidelity DNA polymerase (NEB, USA) with primer pairs of MgActinF + MgActinR, BAS1-F + BAS1-R, and PWL2-F + PWL2-R (**Table S6**). Thermocycling conditions included initial denaturation at 98˚C for 2 min, 25 cycles of 98˚C for 8 s, 63˚C for 30 s and 72˚C for 30 s, and final extension at 72˚C for 5 min.

qRT-PCR

Infected wheat leaf tissues were obtained from 9-day old wheat seedlings (c.v. Bob White), inoculated with B71 (5x10^4^ spores/mL) and harvested 36 hours post inoculation. B71 mycelia were obtained from liquid cultures (3-3-3 media) grown in flasks on a rotating shaker (100 rpm; 25˚C) for 5 days. Total RNA from mycelium and infected wheat leaf tissues were extracted using miVana RNA isolation kit (Invitrogen) and pretreated with DNAse I amplification grade (1 U/μl) (Invitrogen Inc, Carlsbad, CA) according to manufacturer's instructions. RNA quantity and quality were assessed, and cDNA was synthesized using 1 μg of total RNA extracted from each sample. RNA was reverse transcribed with 1 mM of Oligo (dT)_20_, 0.5 mM dNTP mix and 1 μL Superscript III reverse transcriptase (200 U/μl) (Invitrogen, Carlsbad, CA) in a final volume of 20 μL following manufacturer’s instructions. qRT-PCR was performed in a total volume of 10 µL, with 100 ng of total cDNA, using the IQ^TM^ SYBR^®^ Green Super Mix reagent (BioRad, Hercules, CA) according to manufacturer instructions. The *M. oryzae* actin gene (MGG_03982) was amplified using MgActinF and MgActinR primers. Primers MgPwl2F and Pwl2_qRT2-R4 were used to amplify the *PWL2* gene. Primers MgBas1F and MgBas1R were used to amplify the *BAS1* gene. Melting curve and agarose gel analyses confirmed amplification of a single product. Cycle threshold values (*C_t_*) of three independent replicates were used to quantify gene expression. Relative expression (RR) levels of each gene of interest (goi) was calculated and normalized using the *actin* gene as reference [12,28] $(RR=100\times2^{(C_{t}actin-C_{t}goi)})$. For$C_{t}$values of NA (Not Available), the value of 40 cycles was used.

Identification of transposable elements

Transposable element DNA was identified using a combination of programs. Repeats were identified *de novo* using MGEScan [29], LTR_Finder [30] and the LTRharvest program from GenomeTools [31,32]. Consensus Long-Terminal Repeat retrotransposons (LTR-RT) were identified from the output of these three programs using LTR_retriever [33]. Putative Non-LTR retrotransposons identified from MGEScan were validated using BLASTx against a database containing reverse-transcriptase (RT) and transposase (DDE) domains. Elements containing significant similarity to either the RT or DDE1 domain, but not both, were kept and classified as either Long Interspersed Nuclear Elements (LINE) or a DNA transposon. Miniature Inverted-repeats were identified using MITE-hunter [34]. Putative MITEs containing multiple insertions in the genome were manually checked for the presence of terminal inverted repeat (TIR) domains and nucleotide divergence flanking either side of the elements. The LTR, LINE, DNA transposon and MITE elements were combined into a single database (intermediate DB) and clustered using the CD-HIT software. The resulting non-redundant database was used to mask repeats in the B71 assembly with RepeatMasker (Smit AFA, 2013-2015). An additional round of *de novo* repeat identification was carried out on the masked genome using RepeatModeler (Smit AFA, 2008-2015). Previously characterized repeats corresponding to *M. oryzae* from the RepBase database were also identified [35]. The intermediate DB was combined with the RepeatModeler and RepBase repeats, and again clustered to produce a non-redundant database, which was served as a custom repeat library to identify repeats in the B71 genome using RepeatMasker.

Analysis of RIP-type polymorphisms

Based on the RepeatMasker result, genomic sequences of the most abundant transposon elements were extracted and aligned to corresponding transposon sequences from the RepeatMasker database as the reference sequences. The GC percentages of the reference sequences were from 41.3% to 59.3%, which were used to examine relative RIP levels of queries. Only alignments with >60% coverage of a query that is the sequence from the genome were retained. Polymorphisms of each sequence were extracted from the alignments and types of mismatches were determined. The nucleotide changes of C-T or G-A from the reference sequence to the query were considered RIP-type variants. For each transposon sequence, the null hypothesis of that the mean of proportions of RIP-type variants out of the total mismatches transposons located at core chromosomes was not different with that of transposons located at the mini-chromosome was statistically tested using t-test.

For two long transposon elements, MGR583 and POT2, intact homologs were extracted from core chromosomes and the mini-chromosome. The levels of RIP-type mutations of individual sequences were also determined.

**References**

1. Zhang MP, Zhang Y, Scheuring CF, Wu CC, Dong JJ, et al. (2012) Preparation of megabase-sized DNA from a variety of organisms using the nuclei method for advanced genomics research. Nature Protocols 7: 467-478.

2. Clarke JD (2009) Cetyltrimethyl ammonium bromide (CTAB) DNA miniprep for plant DNA isolation. Cold Spring Harb Protoc 2009: pdb.prot5177.

3. Myers EW, Sutton GG, Delcher AL, Dew IM, Fasulo DP, et al. (2000) A whole-genome assembly of Drosophila. Science 287: 2196-2204.

4. Koren S, Walenz BP, Berlin K, Miller JR, Bergman NH, et al. (2017) Canu: scalable and accurate long-read assembly via adaptive k-mer weighting and repeat separation. Genome Res 27: 722–736.

5. Bolger AM, Lohse M, Usadel B (2014) Trimmomatic: a flexible trimmer for Illumina sequence data. Bioinformatics 30: 2114-2120.

6. Weisenfeld NI, Yin S, Sharpe T, Lau B, Hegarty R, et al. (2014) Comprehensive variation discovery in single human genomes. Nat Genet 46: 1350-1355.

7. Li H, Durbin R (2010) Fast and accurate long-read alignment with Burrows-Wheeler transform. Bioinformatics 26: 589-595.

8. McKenna A, Hanna M, Banks E, Sivachenko A, Cibulskis K, et al. (2010) The Genome Analysis Toolkit: a MapReduce framework for analyzing next-generation DNA sequencing data. Genome Res 20: 1297-1303.

9. Li H (2011) A statistical framework for SNP calling, mutation discovery, association mapping and population genetical parameter estimation from sequencing data. Bioinformatics 27: 2987-2993.

10. Boetzer M, Henkel CV, Jansen HJ, Butler D, Pirovano W (2011) Scaffolding pre-assembled contigs using SSPACE. Bioinformatics 27: 578-579.

11. Grabherr MG, Haas BJ, Yassour M, Levin JZ, Thompson DA, et al. (2011) Full-length transcriptome assembly from RNA-Seq data without a reference genome. Nature Biotechnology 29: 644-U130.

12. Mosquera G, Giraldo MC, Khang CH, Coughlan S, Valent B (2009) Interaction transcriptome analysis identifies *Magnaporthe oryzae* BAS1-4 as biotrophy-associated secreted proteins in rice blast disease. Plant Cell 21: 1273-1290.

13. Dobin A, Davis CA, Schlesinger F, Drenkow J, Zaleski C, et al. (2013) STAR: ultrafast universal RNA-seq aligner. Bioinformatics 29: 15-21.

14. Love MI, Huber W, Anders S (2014) Moderated estimation of fold change and dispersion for RNA-seq data with DESeq2. Genome Biol 15: 550.

15. Benjamini Y, Hochberg Y (1995) Controlling the False Discovery Rate - a Practical and Powerful Approach to Multiple Testing. Journal of the Royal Statistical Society Series B-Methodological 57: 289-300.

16. Cantarel BL, Korf I, Robb SM, Parra G, Ross E, et al. (2008) MAKER: an easy-to-use annotation pipeline designed for emerging model organism genomes. Genome Res 18: 188-196.

17. Salamov AA, Solovyev VV (2000) Ab initio gene finding in Drosophila genomic DNA. Genome Res 10: 516-522.

18. Jones P, Binns D, Chang HY, Fraser M, Li W, et al. (2014) InterProScan 5: genome-scale protein function classification. Bioinformatics 30: 1236-1240.

19. Parra G, Bradnam K, Ning Z, Keane T, Korf I (2009) Assessing the gene space in draft genomes. Nucleic Acids Res 37: 289-297.

20. Nielsen H (2017) Predicting secretory proteins with SignalP. Methods Mol Biol 1611: 59-73.

21. Young MD, Wakefield MJ, Smyth GK, Oshlack A (2010) Gene ontology analysis for RNA-seq: accounting for selection bias. Genome Biol 11: R14.

22. Paradis E, Claude J, Strimmer K (2004) APE: analyses of phylogenetics and evolution in R language. Bioinformatics 20: 289-290.

23. Marcais G, Kingsford C (2011) A fast, lock-free approach for efficient parallel counting of occurrences of k-mers. Bioinformatics 27: 764-770.

24. Olshen AB, Venkatraman ES, Lucito R, Wigler M (2004) Circular binary segmentation for the analysis of array-based DNA copy number data. Biostatistics 5: 557-572.

25. Orbach MJ, Chumley FG, Valent B (1996) Electrophoretic karyotypes of Magnaporthe grisea pathogens of diverse grasses. Mol Plant Microbe Interact 9: 261-271.

26. Orbach MJ, Vollrath D, Davis RW, Yanofsky C (1988) An electrophoretic karyotype of Neurospora-Crassa. Molecular and Cellular Biology 8: 1469-1473.

27. Xue M, Yang J, Li Z, Hu S, Yao N, et al. (2012) Comparative analysis of the genomes of two field isolates of the rice blast fungus Magnaporthe oryzae. PLoS Genet 8: e1002869.

28. Liu S, Dietrich CR, Schnable PS (2009) DLA-based strategies for cloning insertion mutants: cloning the gl4 locus of maize using Mu transposon tagged alleles. Genetics 183: 1215-1225.

29. Lee H, Lee M, Mohammed Ismail W, Rho M, Fox GC, et al. (2016) MGEScan: a Galaxy-based system for identifying retrotransposons in genomes. Bioinformatics 32: 2502-2504.

30. Xu Z, Wang H (2007) LTR_FINDER: an efficient tool for the prediction of full-length LTR retrotransposons. Nucleic Acids Res 35: W265-268.

31. Ellinghaus D, Kurtz S, Willhoeft U (2008) LTRharvest, an efficient and flexible software for de novo detection of LTR retrotransposons. BMC Bioinformatics 9: 18.

32. Gremme G, Steinbiss S, Kurtz S (2013) GenomeTools: a comprehensive software library for efficient processing of structured genome annotations. IEEE/ACM Trans Comput Biol Bioinform 10: 645-656.

33. Ou S, Jiang N (2018) LTR_retriever: a highly accurate and sensitive program for identification of long terminal-repeat retrotransposons. Plant Physiol 17: 1410-1422.

34. Han Y, Wessler SR (2010) MITE-Hunter: a program for discovering miniature inverted-repeat transposable elements from genomic sequences. Nucleic Acids Res 38: e199.

35. Bao W, Kojima KK, Kohany O (2015) Repbase Update, a database of repetitive elements in eukaryotic genomes. Mob DNA 6: 11.

36. Tosa Y, Hirata K, Tamba H, Nakagawa S, Chuma I, et al. (2004) Genetic Constitution and Pathogenicity of Lolium Isolates of Magnaporthe oryzae in Comparison with Host Species-Specific Pathotypes of the Blast Fungus. Phytopathology 94: 454-462.

37. Farman M, Peterson GL, Chen L, Starnes JH, Valent B, et al. (2017) The *Lolium* pathotype of *Magnaporthe oryzae* recovered from a single blasted wheat plant in the United States. Plant Disease 101: 684-692.

38. Pieck ML, Ruck A, Farman ML, Peterson GL, Stack JP, et al. (2017) Genomics-based marker discovery and diagnostic assay development for wheat blast. Plant Disease 101: 103-109.

39. Gladieux P, Condon B, Ravel S, Soanes D, Nunes Maciel JL, et al. (2018) Gene flow between divergent cereal- and grass-specific lineages of the rice blast fungus *Magnaporthe oryzae*. mBio 9:e01219-17.

40. Malaker PK, Barma NCD, Tiwari TP, W.J.Collis WJ, Duveiller E, et al. (2016) First report of wheat blast caused by Magnaporthe oryzae pathotype triticum in Bangladesh. Plant Disease 100: 2330
