# Supplementary figures and images for: Effector gene reshuffling involves dispensable mini-chromosomes in the wheat blast fungus
Source: PLoS Genet. 2019 Sep 12;15(9):e1008272. doi: 10.1371/journal.pgen.1008272 (PMC6741851; doi:10.1371/journal.pgen.1008272)

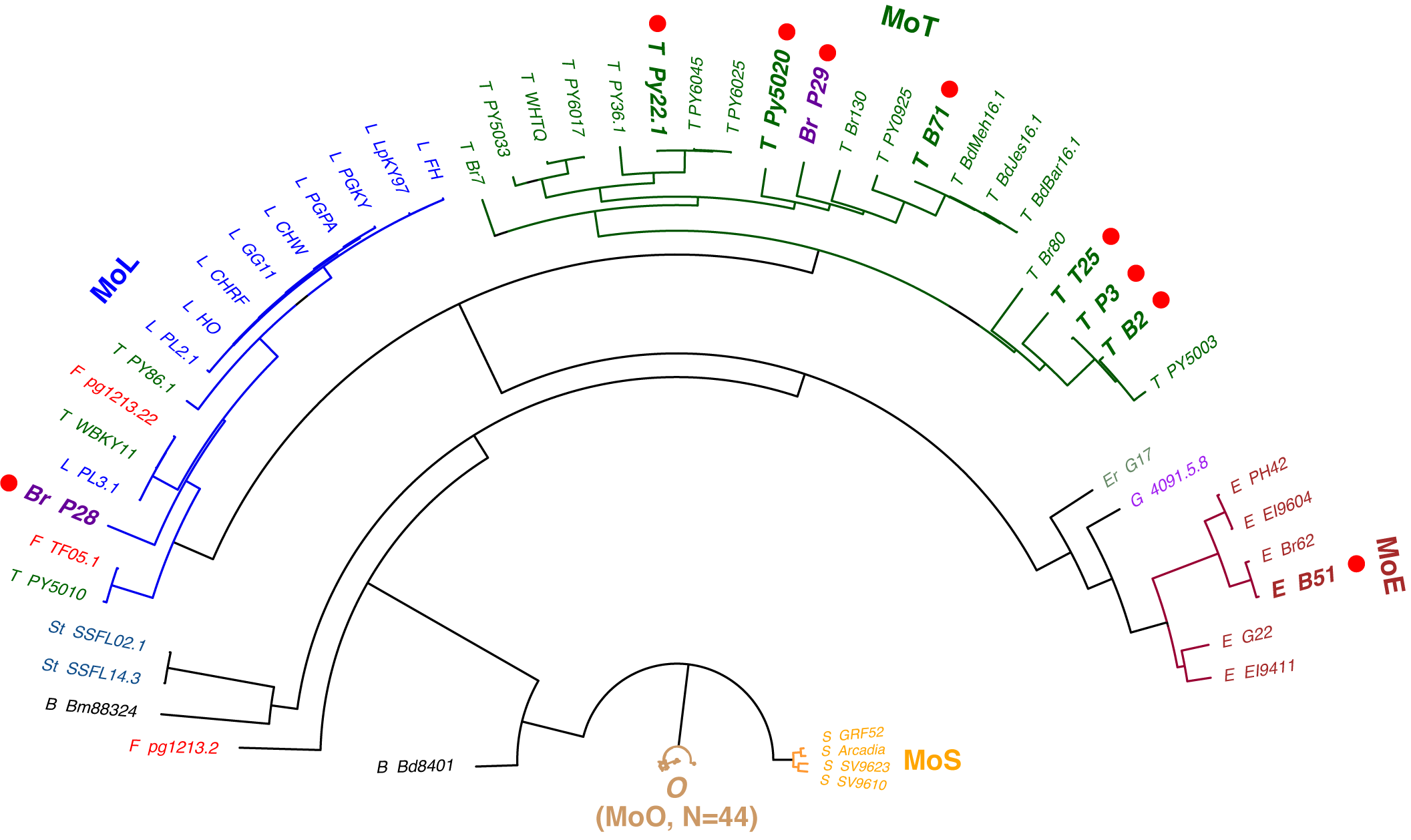

Supplement: S1 Fig — These are: Oryza pathotype (MoO, 44 Strains); Setaria pathotype (MoS, 4 strains); Eleusine pathotype (MoE, 6 strains); Triticum pathotype (MoT, 21 strains); and Lolium pathotype (MoL, 16 strains). Strain branches in each of five pathotypes were labeled with the same color as the pathotype identifier. Assembly data of each strain were utilized to identify polymorphisms and construct the phylogeny with the neighbor-joining tree estimation. Strains selected in this study are highlighted with red dots. Host species on which each strain was isolated from the field are indicated (e.g., T) by: B, Brachiaria; Br, Bromus; E, Eleusine; Er, Eragrostis; F, Festuca; L, Lolium; O, Oryza; S, Setaria; St, Stenotaphrum; T, Triticum. The strain G 4091-5-8, which infects both Eragrostis spp. and Eleusine spp., was obtained in a laboratory cross between E G22 and Er G17. Strains Py22.1 and Py5020 are described in Pieck et al, 2017; and all other non-MoO strains are described in Gladieux et al, 2018. (TIF) [file pgen.1008272.s002.tif]

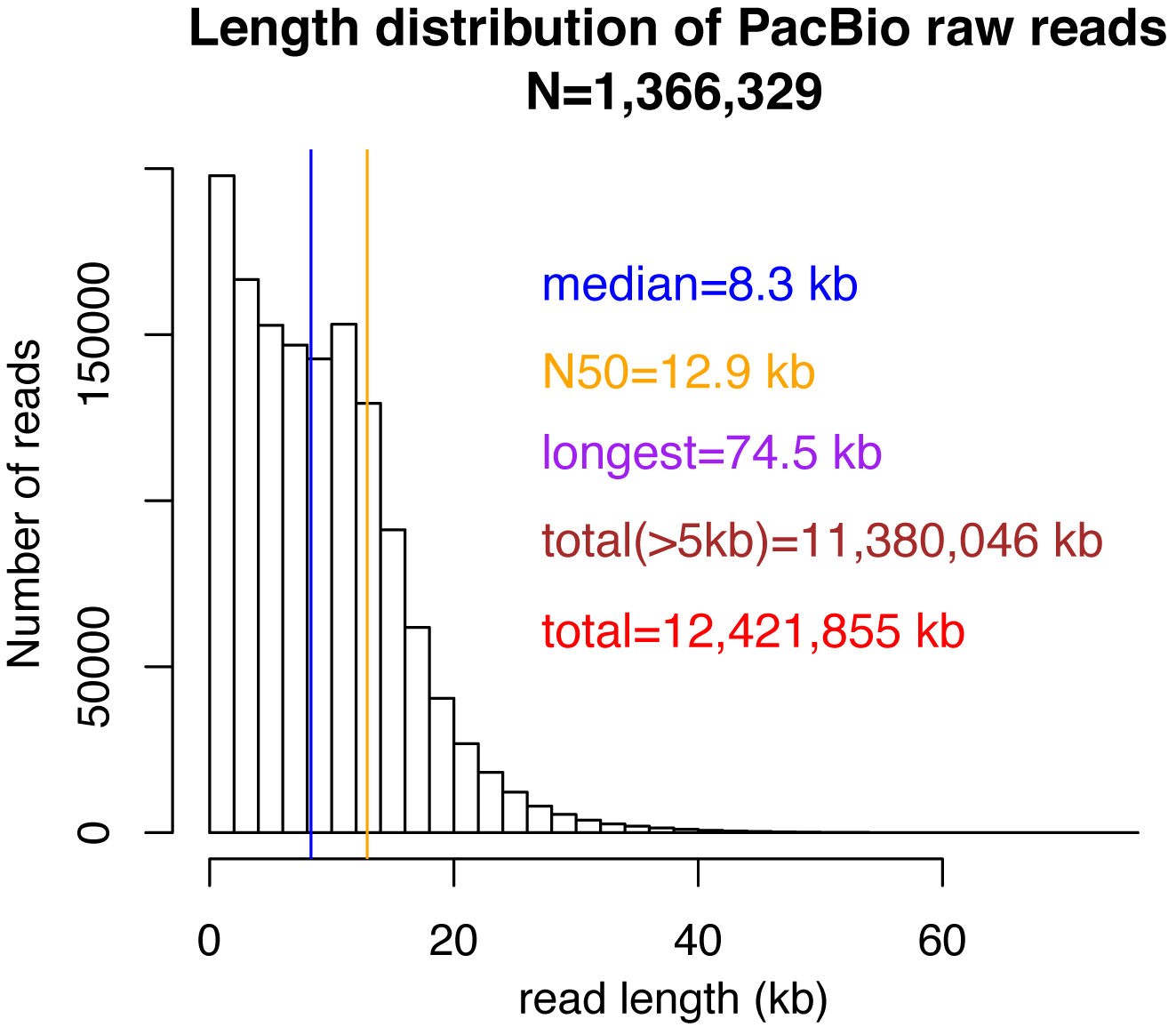

Supplement: S2 Fig — The number of reads, the median length, the N50, the longest length, the total length of reads greater than 5 kb, and the total length of all reads were reported. The median length and the N50 are indicated with blue and orange vertical lines, respectively. During the Canu assembly, only reads with the minimum of 14,378 bp were extracted for read correction (first step of Canu assembly). When the Quiver error correction was performed, all raw reads were used. (TIF) [file pgen.1008272.s003.tif]

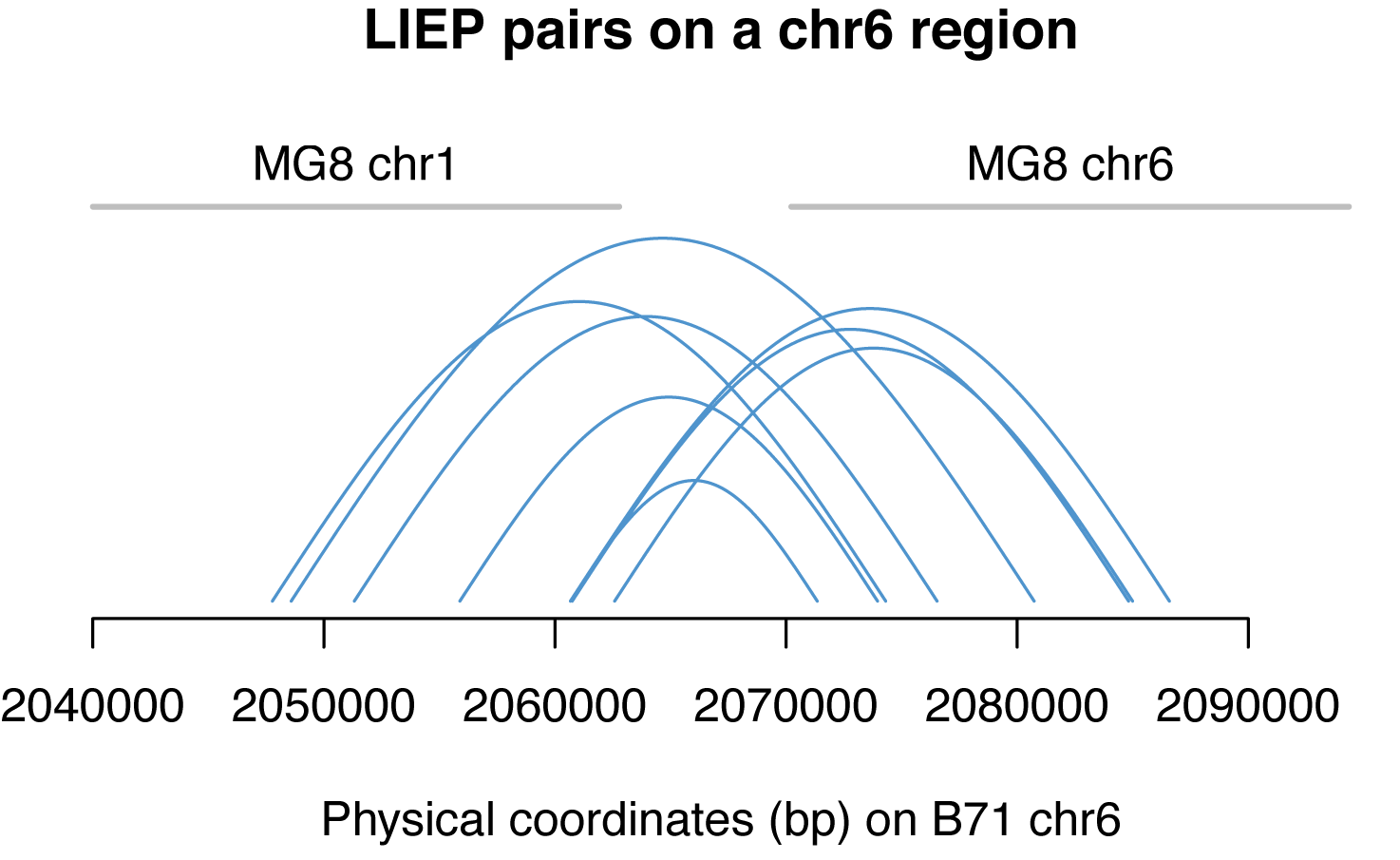

Supplement: S3 Fig — The region (chromosome 6, 1–2,062,779 bp) of B71Ref1 is collinear with a partial sequence of MG8 chromosome 1, and the region beyond 2,070,228 bp of B71Ref1 chromosome 6 is collinear with MG8 chromosome 6. Blue curves showed pairs of LIEP sequences spanning the junction region, from 2,062,779 bp to 2,070,228 bp. In addition, the junction region and some flanking sequences are fully covered by 50 single PacBio long reads. (TIF) [file pgen.1008272.s004.tif]

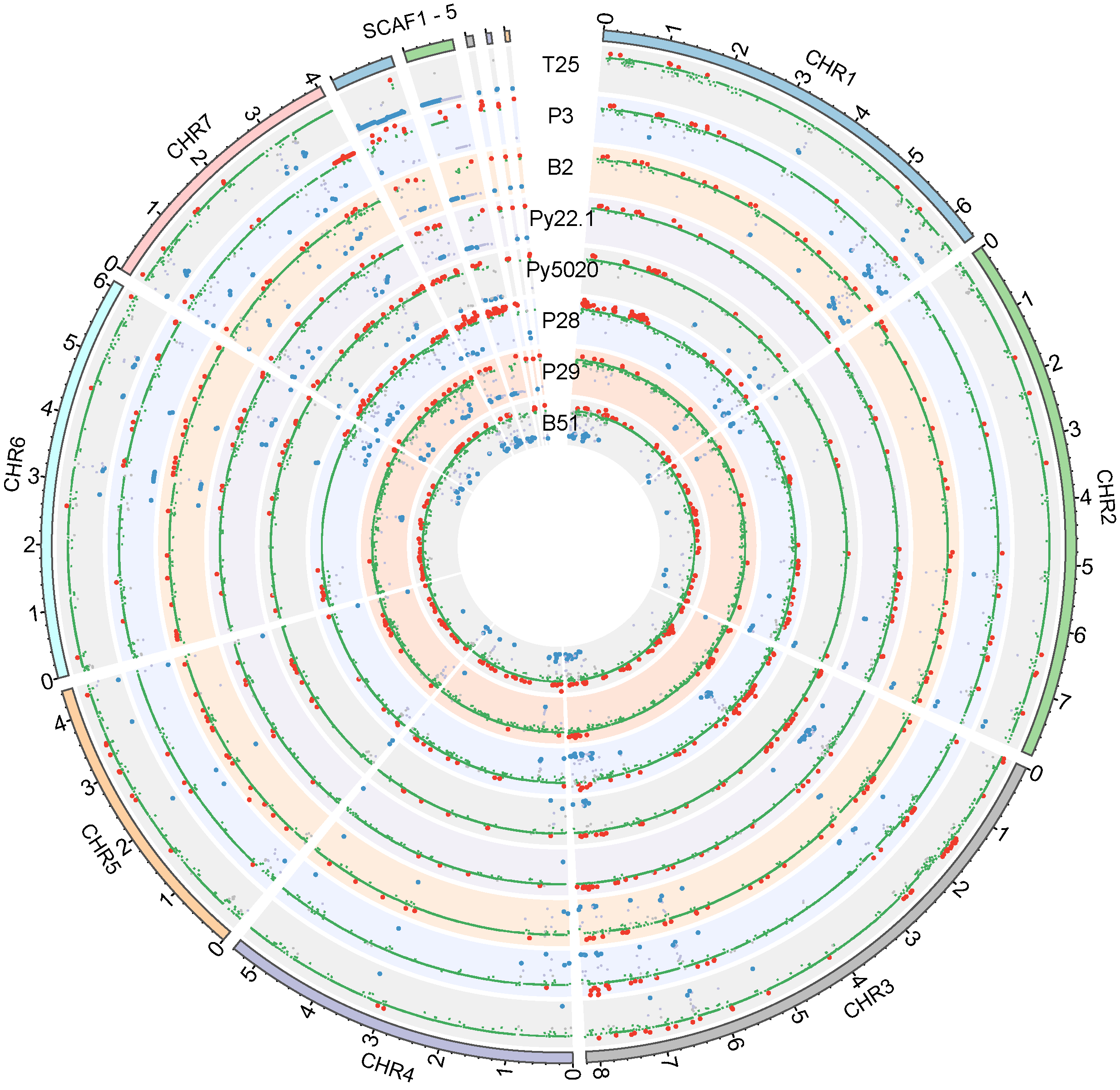

Supplement: S4 Fig — The strains being compared are MoT strains T25, P3, B2, Py22.1, Py5020 and P29; the MoL strain P28; and the MoE strain B51. Each track represents a copy number comparison of the non-B71 isolate versus B71. The value of CNV index, which represents the log2 value of the ratio of sequencing read counts in genomic segments between two isolates of the comparison, determines vertical position on the track. Red, blue, green lines represent CNplus, CNminus, and CNequal regions relative to the B71. (TIF) [file pgen.1008272.s005.tif]

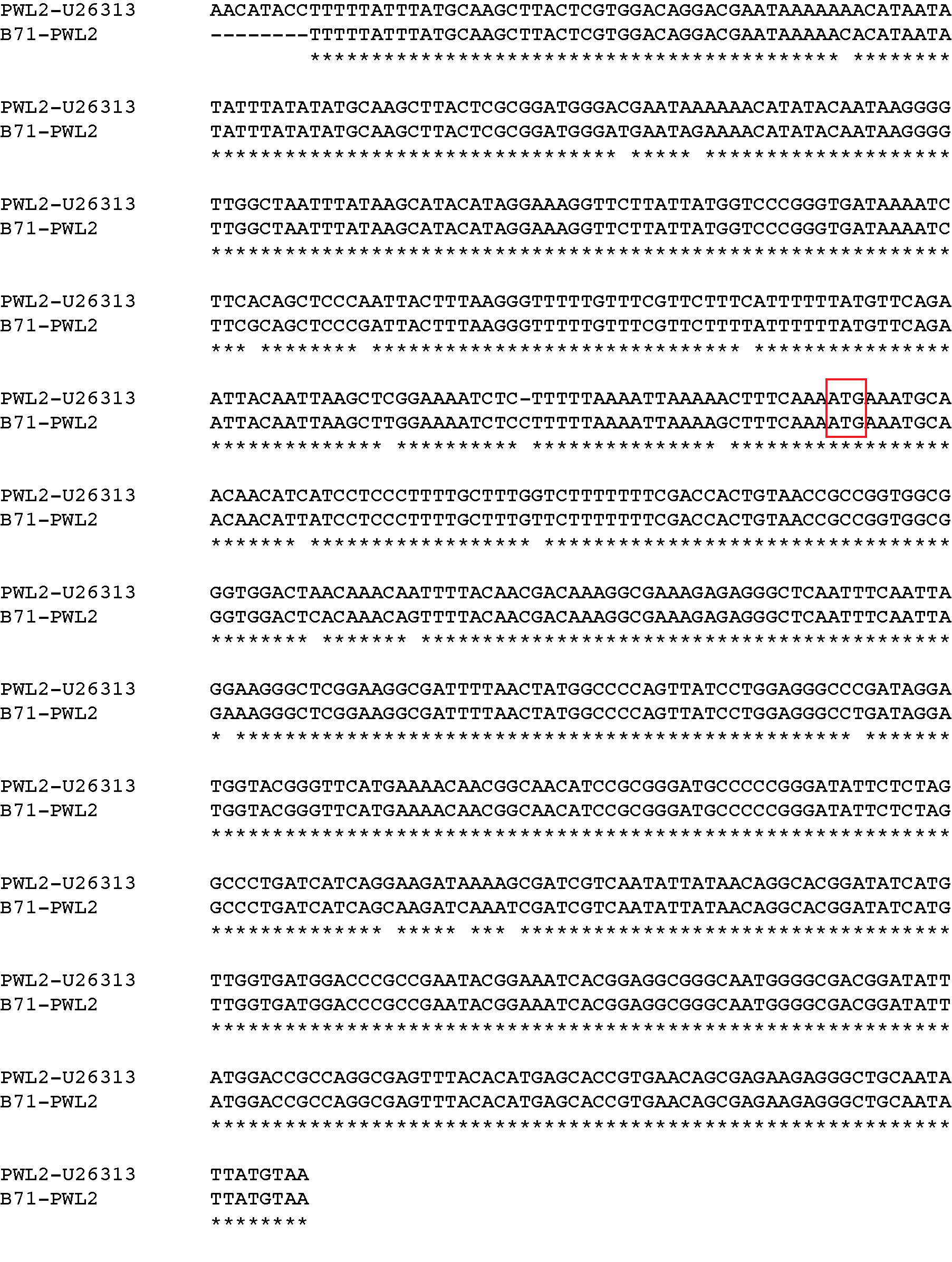

Supplement: S5 Fig — PWL2-U26313 is a partial sequence of the Genbank accession U26313. The translation start site is highlighted in a red box. (TIF) [file pgen.1008272.s006.tif]

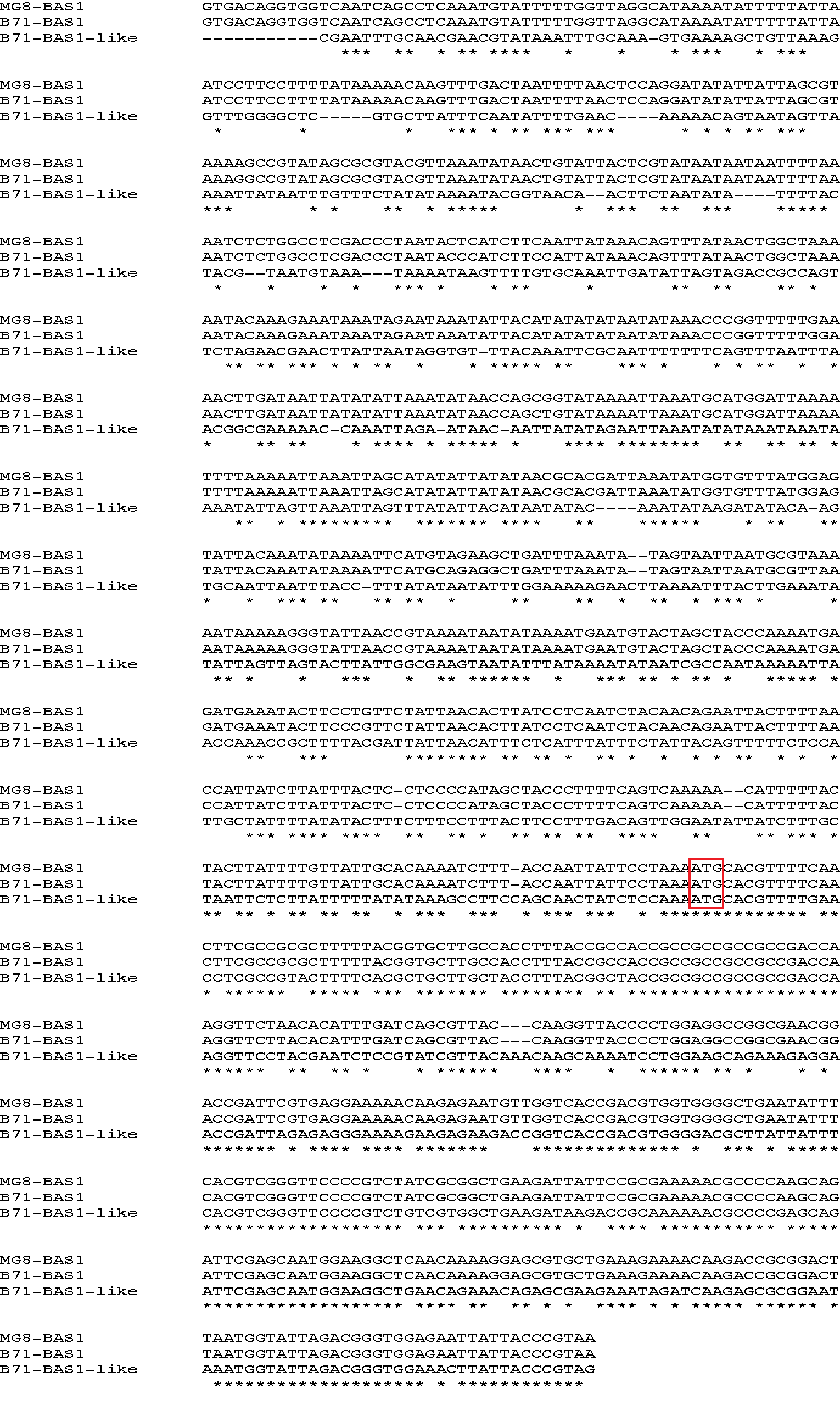

Supplement: S6 Fig — Two BAS1 homologs were aligned with the BAS1 from 70–15. The homolog from B71 chromosome 1 (BAS1-chr1) has <70% identity with BAS1 of 70–15. MG8-BAS1 is a partial sequence of the Genbank accession FJ807764.1. The translation start site is highlighted in a red box. (TIF) [file pgen.1008272.s007.tif]

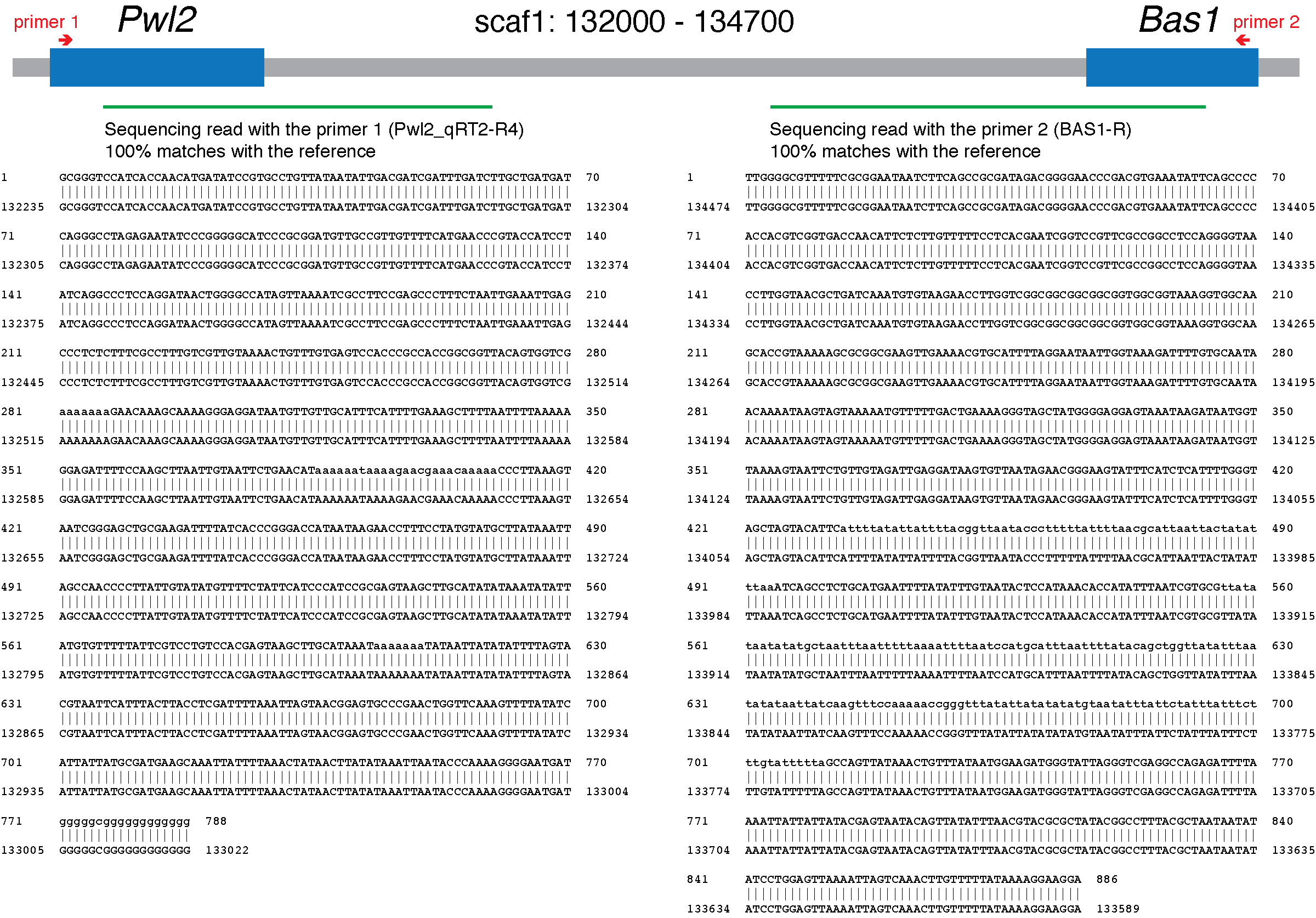

Supplement: S7 Fig — Validation of the neighboring structure of PWL2 and BAS1 via Sanger sequencing. The PCR product using the primers Pwl2_qRT2-R4 (primer 1) and BAS1-R (primer 2) was sequenced using these two primers separately. Green lines indicate the alignment regions on the scaf1 for two sequencing reads. Detailed alignments of two sequencing reads were shown underneath each green line. (TIF) [file pgen.1008272.s008.tif]

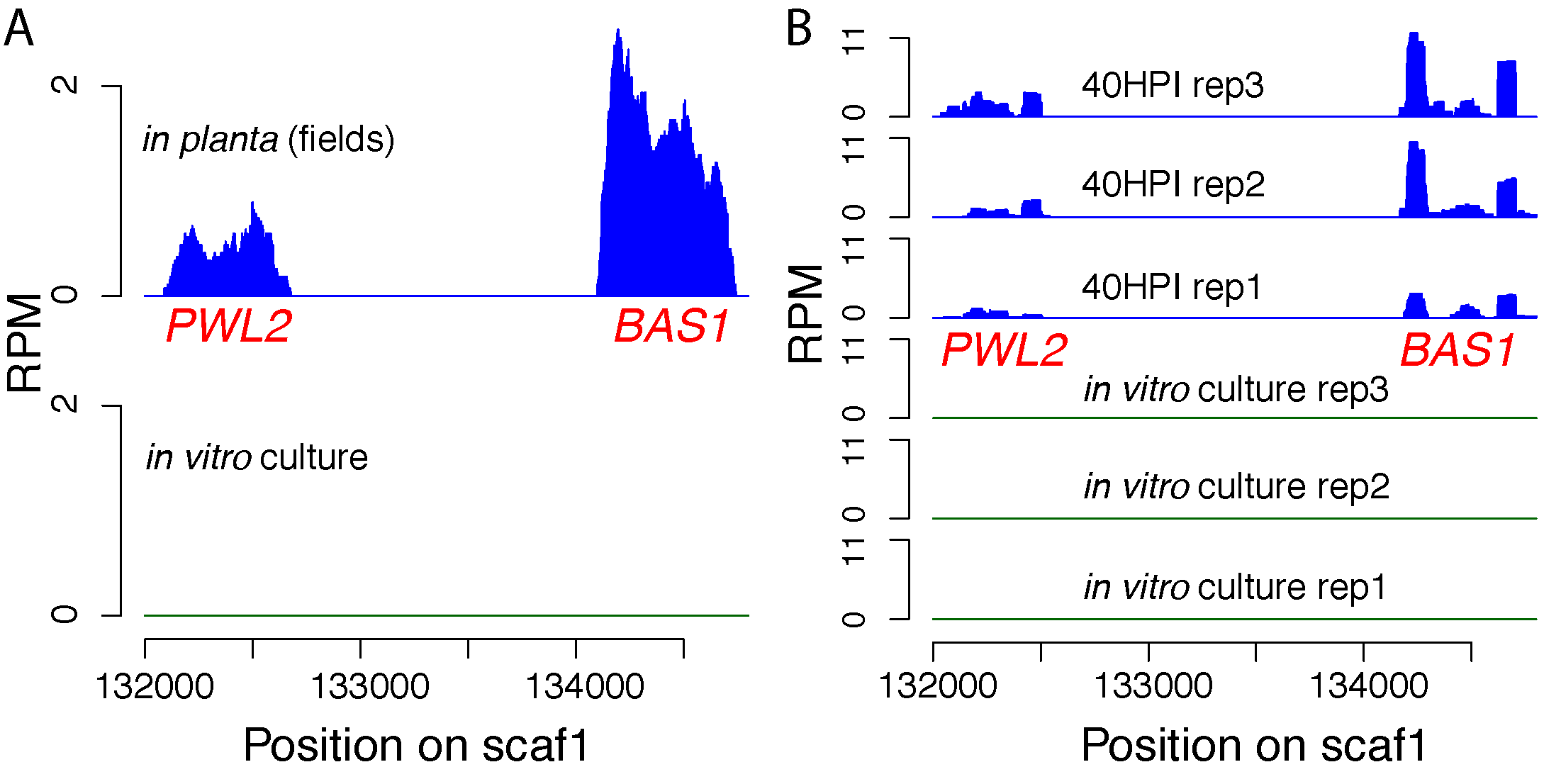

Supplement: S8 Fig — RNA-Seq reads distribution of PWL2 and BAS1. A) Distributions of uniquely mapped reads from the field samples (2x101bp paired end data) and in vitro culture samples (2x150bp paired-end data) from the first RNA-Seq experiment. B) Distribution of uniquely mapped 75bp reads from three biological replicates of 40 HPI in planta B71 samples and three biological replicates of in vitro cultured B71 samples from the second RNA-Seq experiment. Note that shapes of read distributions are related to read lengths that influence mapping ability of reads. RPM (reads per million of total aligned reads) represents normalized read counts. (TIF) [file pgen.1008272.s009.tif]

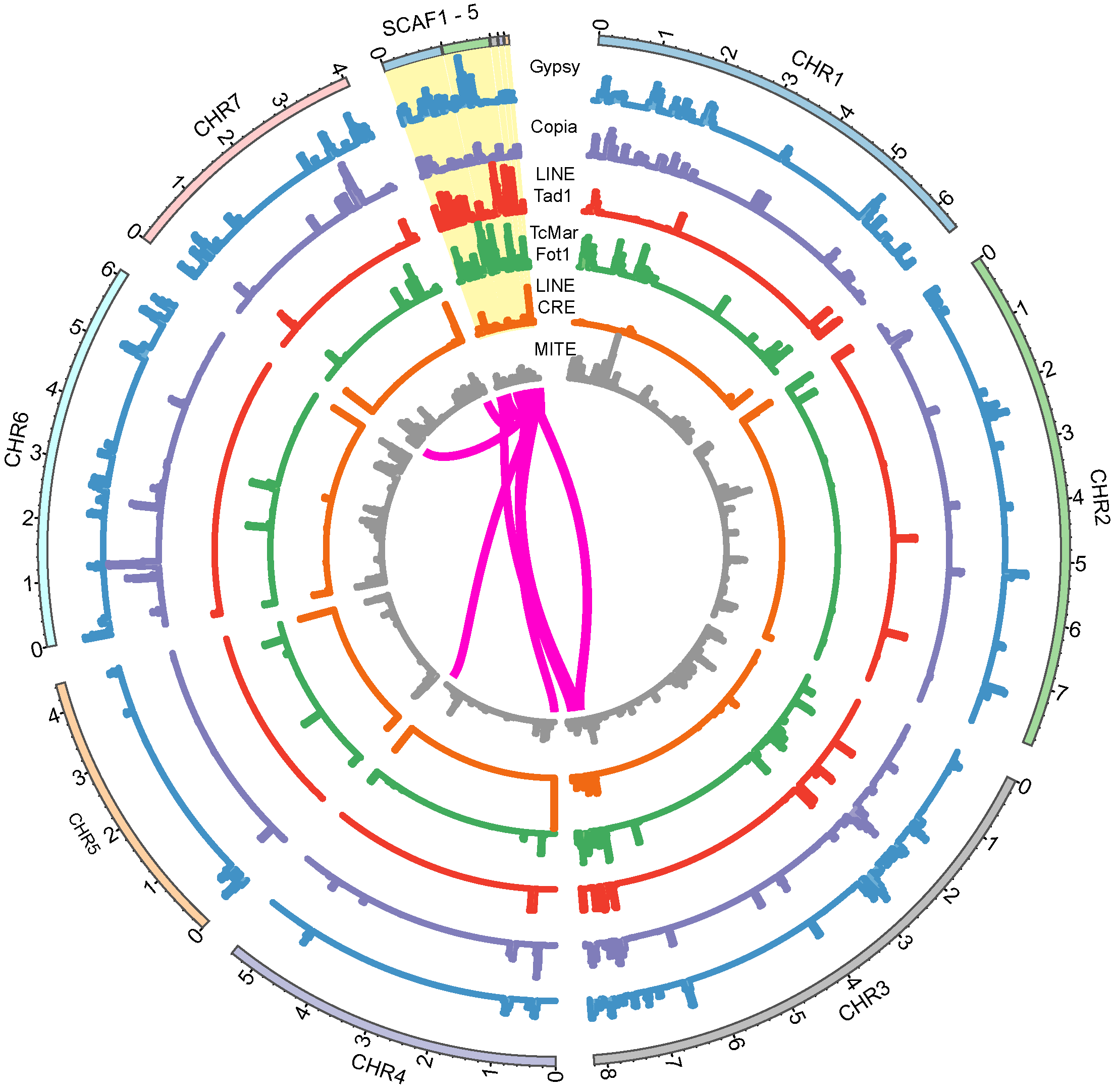

Supplement: S9 Fig — The inset in the center shows large genome duplications (>10 kb and >95% identity) within five scaffolds, as well as between the scaffolds and the chromosomes 1–7. LINE subclass Tad1 contains the previously characterized retrotransposon MGR583 and subclass CRE contains the telomere-targeted retrotransposon MoTeR. The DNA transposon subclass TcMar-Fot1 contains previously characterized Pot2. (TIF) [file pgen.1008272.s010.tif]

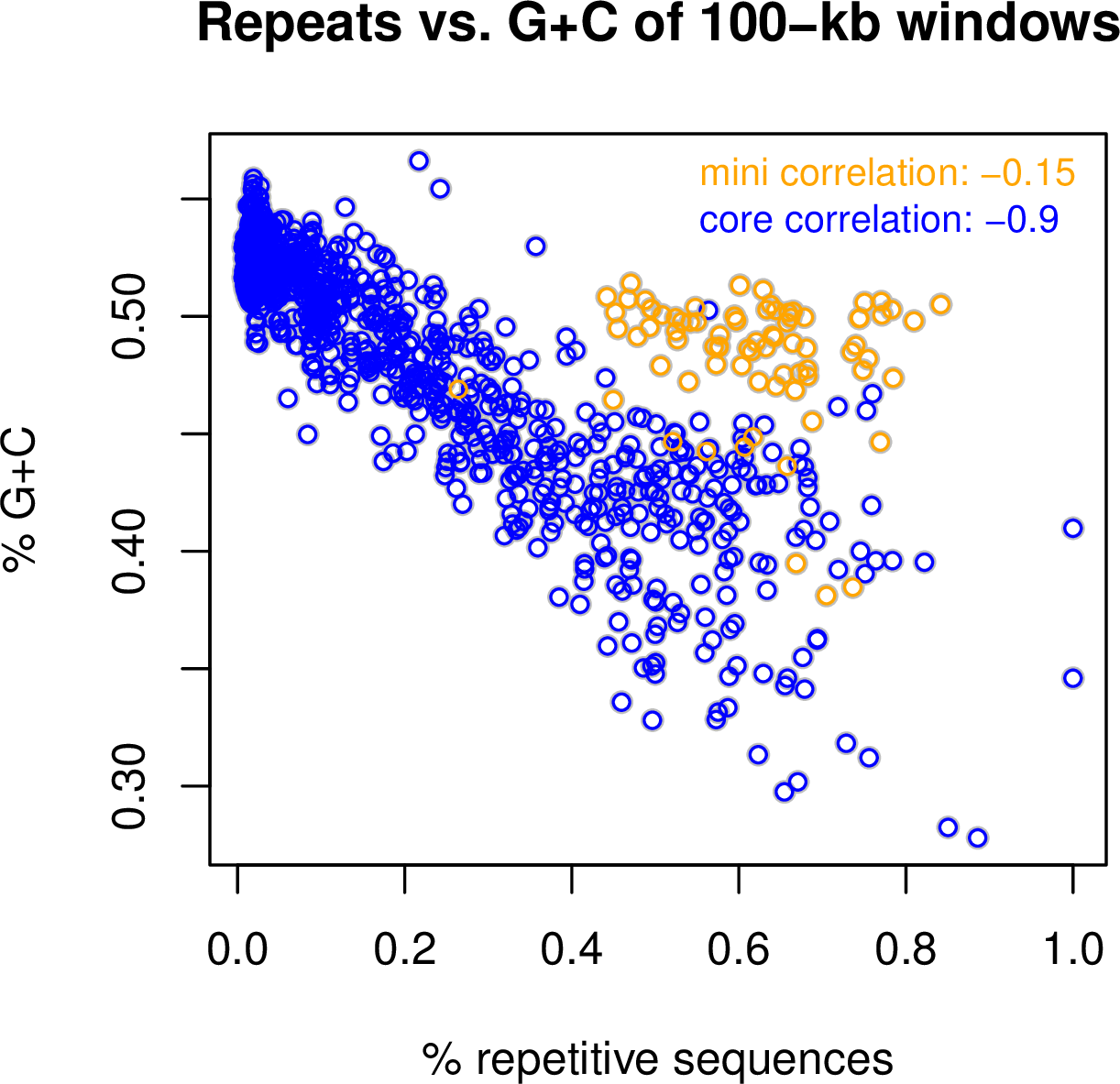

Supplement: S10 Fig — Orange and blue circles represent 100-kb bins from the B71 mini-chromosome (five scaffolds) and core chromosomes, respectively. Pearson correlations between GC percentages and proportions of repetitive sequences of 100-kb non-overlap genomic bins of the mini-chromosome and core chromosomes. (TIF) [file pgen.1008272.s011.tif]

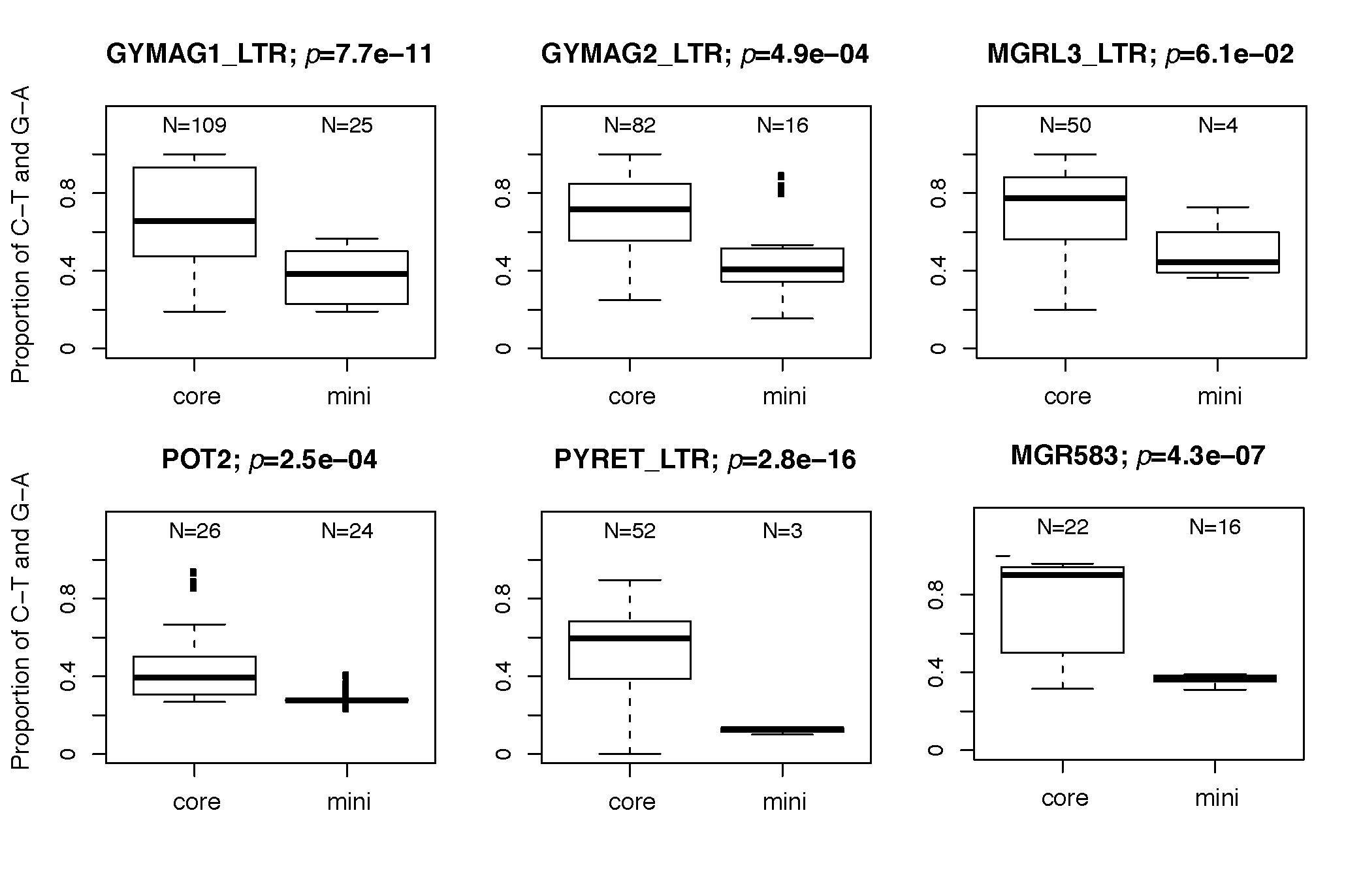

Supplement: S11 Fig — Genomic sequences of each transposable element were aligned to corresponding transposon sequences from the RepeatMasker database as the reference sequences. Polymorphisms were determined for each sequence that exhibits at least 60% overlap with the reference sequence. For each transposon element, a t-test was performed to test the null hypothesis that the mean proportions of RIP-type variants out of the total mismatches of transposons located in core chromosomes was not different from that of transposons located at the mini-chromosome. P-values of t-tests were shown on the top of each boxplot. (TIF) [file pgen.1008272.s012.tif]

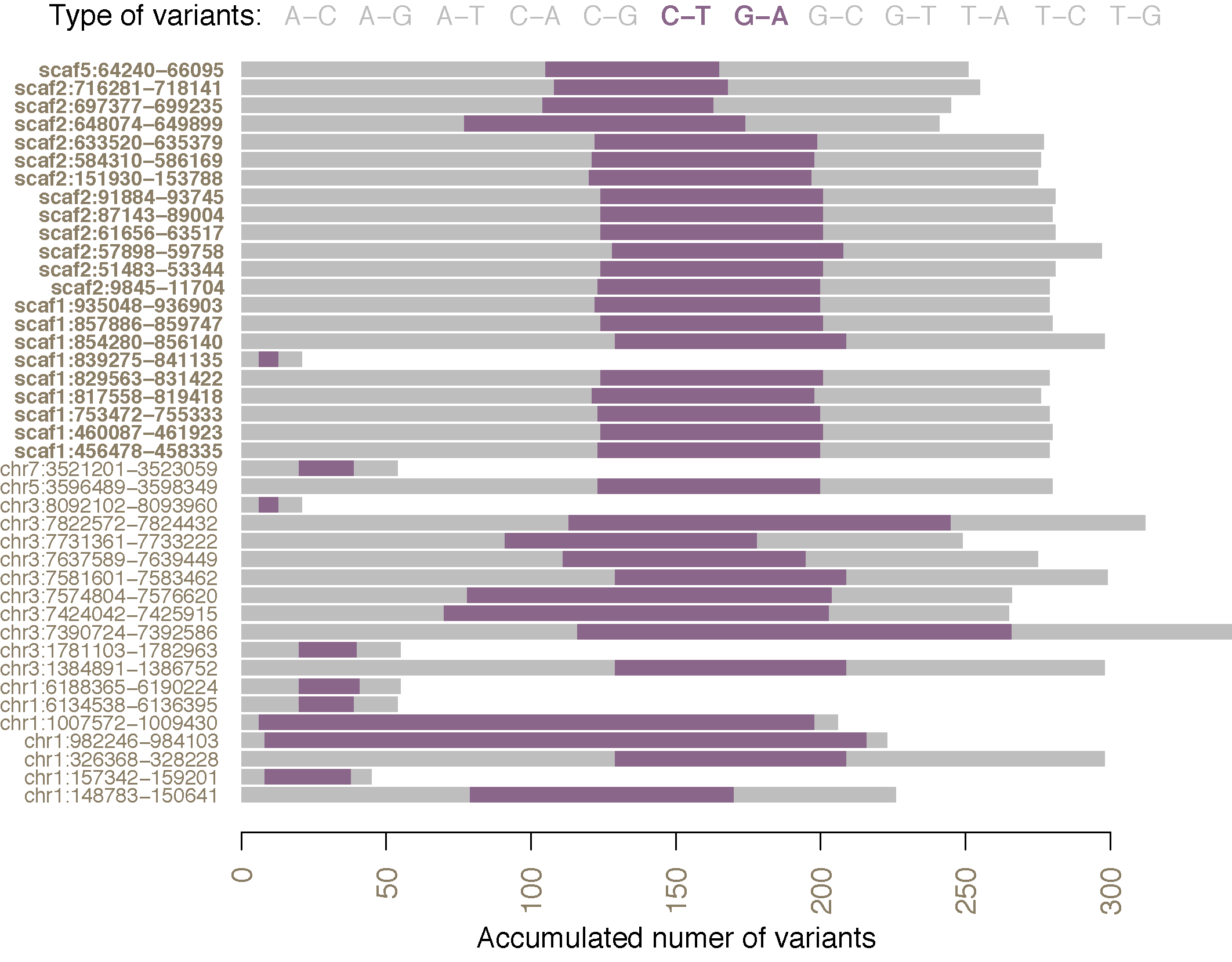

Supplement: S12 Fig — Sequences of Pot2 homologs were aligned with the reference Pot2. Mismatching variants of each Pot2 homolog relative to the reference Pot2 were categorized based on nucleotide changes. All twelve variant types were listed on the top. For example, A-C represents base A on the reference Pot2 is changed to base C on Pot2 homologs. Each row shows the accumulated number of variants of a Pot2 homolog at the order of type of variation listed on the top. Two RIP-type mutations were highlighted in purple. Labels on the left show genomic locations of each Pot2 homolog. (TIF) [file pgen.1008272.s013.tif]

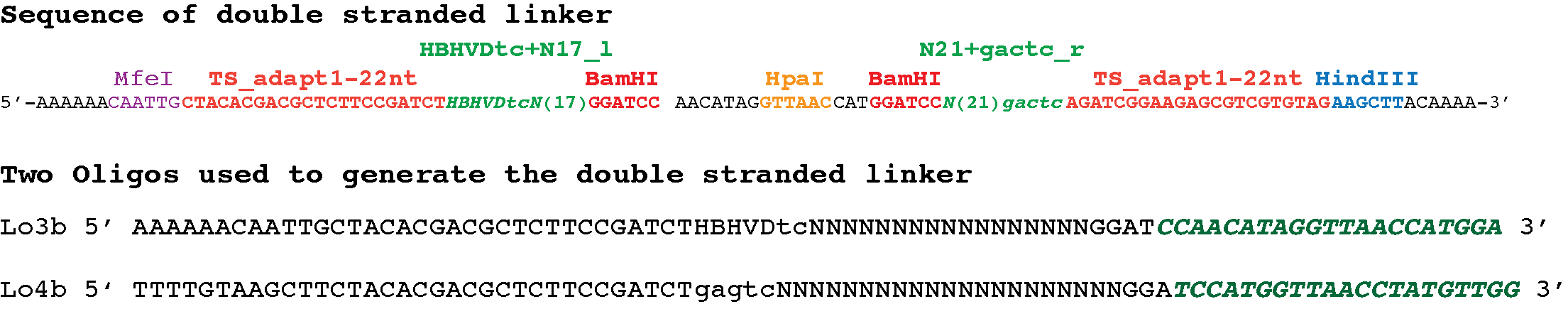

Supplement: S13 Fig — Two synthetic oligos with random barcodes and Illumina compatible sequence were annealed by 21 bp overlapping sequence (green italic sequences of Lo3b and Lo4b). The annealed product was then filled to form a double-stranded linker DNA (top sequence). The design of the link was shown. N(17) and N(21) indicated 17 and 21 randomly synthesized nucleotides, respectively. The linker sequence contains other IUPAC nucleotide code (e.g., H = A, C or T). (TIF) [file pgen.1008272.s014.tif]
