# Supplementary material for: Effector gene reshuffling involves dispensable mini-chromosomes in the wheat blast fungus
Source: PLoS Genet. 2019 Sep 12;15(9):e1008272. doi: 10.1371/journal.pgen.1008272 (PMC6741851; doi:10.1371/journal.pgen.1008272)
Supplement: S1 Table — (DOCX) [file pgen.1008272.s015.docx]

**S1 Table**. Strains used in this study

| Name | Isolation Host | Mo pathotype (wheat pathogenicity^1^) | Year isolated | Isolation location | Genome Accession | Reference |
| --- | --- | --- | --- | --- | --- | --- |
| B2 | Wheat | MoT (+++) | 2011 | Quirusillas, Bolivia | MDUN00000000 | [1] |
| B51 | Goosegrass^2^ (*Eleusine indica*) | MoE (-) | 2012 | Quirusillas, Bolivia |  |  |
| B71 | Wheat | MoT (++++) | 2012 | Okinawa, Bolivia | LXOQ01000000 | [2] |
| P3 | Wheat | MoT (++++) | 2012 | Canindeyú, Paraguay |  |  |
| P28 | Cheat grass^3^ *(Bromus tectorum)* | MoL (nt) | 2014 | Paraguay | MKZV00000000 | [1] |
| P29 | Cheat grass^3^ *(Bromus tectorum)* | MoT (+++) | 2014 | Paraguay | MLCC00000000 | [1] |
| Py22.1 | Wheat | MoT (nt) | 2007 | Paraná, Brazil | MILZ00000000 | [1] |
| Py5020 | Wheat | MoT (nt) | 2005 | Paraná, Brazil | MKIG00000000 | [1] |
| T25 | Wheat | MoT (++) | 1988 | Paraná, Brazil |  |  |

^1^Relative aggressiveness on wheat heads indicated from lowest to highest by -,+,++,+++,++++, with nt indicating not tested.

^2^Isolated from goosegrass in the wheat blast hot spot, Quirusillas, Bolivia.

^3^Isolated from cheat grass in an infected wheat field in Paraguay.

**References**

1. Pieck ML, Ruck A, Farman ML, Peterson GL, Stack JP, et al. (2017) Genomics-based marker discovery and diagnostic assay development for wheat blast. Plant Disease 101: 103-109.

2. Malaker PK, Barma NCD, Tiwari TP, W.J.Collis WJ, Duveiller E, et al. (2016) First report of wheat blast caused by Magnaporthe oryzae pathotype triticum in Bangladesh. Plant Disease 100: 2330
