# Supplementary material for: Effector gene reshuffling involves dispensable mini-chromosomes in the wheat blast fungus
Source: PLoS Genet. 2019 Sep 12;15(9):e1008272. doi: 10.1371/journal.pgen.1008272 (PMC6741851; doi:10.1371/journal.pgen.1008272)
Supplement: S2 Table — (DOCX) [file pgen.1008272.s016.docx]

**S2 Table**. Statistics of PacBio and Illumina assemblies

| **Item** | **PacBio** | **Illumina** |
| --- | --- | --- |
| Minimum cutoff of contig size (bp) | - | 500 |
| **total contig number** | **31** | **2,924** |
| total contig length (bp) | 44,480,730 | 43,297,828 |
| mean contig length (bp) | 1,434,862 | 14,807 |
| median contig length (bp) | 78,719 | 7,460 |
| SD of contig length (bp) | 436,064 | 369 |
| longest contig (bp) | 7,895,253 | 179,619 |
| shortest contig (bp) | 12,906 | 500 |
| **N50 (bp)** | **5,396,842** | **31,133** |
| L50 | 4 | 394 |
| Contigs>=1kbp | 31 | 2,671 |
| Contigs>=5kbp | 31 | 1,720 |
| Contigs>=10kbp | 31 | 1,234 |
| Contigs>=50kbp | 21 | 166 |
| **overall GC** | **0.498** | **0.499** |
| mean contig GC | 0.471 | 0.496 |
| median contig GC | 0.491 | 0.51 |
| SD of contig GC | 0.018 | 0.001 |
| max contig GC | 0.525 | 0.644 |
| min contig GC | 0.284 | 0.205 |
