# Supplementary material for: Effector gene reshuffling involves dispensable mini-chromosomes in the wheat blast fungus
Source: PLoS Genet. 2019 Sep 12;15(9):e1008272. doi: 10.1371/journal.pgen.1008272 (PMC6741851; doi:10.1371/journal.pgen.1008272)
Supplement: S3 Table — (DOCX) [file pgen.1008272.s017.docx]

**S3 Table.** CNV overlapping effectors genes

| Order | Effector genes | Chr | Position | B2 | B51 | P28 | P29 | P3 | Py22.1 | Py5020 | T25 |
| --- | --- | --- | --- | --- | --- | --- | --- | --- | --- | --- | --- |
| 1 | BAS1^*^ | chr1 | 1,228,470 | % | % | % | % | % | % | % | % |
| 2 | AVR1-CO39^#^ | chr2 | 7,253,033 | % | % | % | % | % | % | % | % |
| 3 | AVR-Pik | chr3 | 1,324,102 | % | % | % | % | % | % | % | % |
| 4 | PWL4 | chr3 | 388,420 | % | % | % | % | % | % | % | % |
| 5 | AVR-Pii | chr3 | 7,735,856 | NA | CNminus | & | % | NA | % | NA | % |
| 6 | AVR-Pi54 | chr4 | 1,079,049 | % | % | % | % | % | % | % | % |
| 7 | BAS2 | chr4 | 5,359,621 | % | % | % | % | % | % | % | % |
| 8 | BAS4 | chr5 | 17,696 | % | % | % | % | % | % | % | % |
| 9 | BAS3 | chr5 | 3,450,049 | % | % | % | % | % | % | % | % |
| 10 | PWT3^#^ | chr5 | 3,632,010 | NA | CNplus | % | % | % | % | % | % |
| 11 | AVR-Pib | chr6 | 6,027,571 | CNplus | CNminus | % | CNplus | CNplus | CNplus | % | % |
| 12 | AVR-Pi9 | chr7 | 2,736,023 | % | % | % | % | % | % | % | % |
| 13 | AVRPiz-t | chr7 | 3,041,628 | % | % | % | % | % | % | % | % |
| 14 | AVR-Pita3 | chr7 | 344,264 | %^*^ | % | NA | CNminus | %^*^ | % | % | %^*^ |
| 15 | AVR-Pik_km_kp | chr7 | 3,846,519 | % | % | % | % | CNplus | % | % | % |
| 16 | PWL2 | scaf1 | 132,123 | & | & | % | CNminus | % | & | % | CNminus |
| 17 | BAS1^**^ | scaf1 | 134,228 | & | & | % | CNminus | % | & | % | CNminus |
| 18 | AVR-Pii | scaf1 | 29,460 | & | NA | NA | & | % | & | CNplus | & |

%: CNequal, conserved between the strain and B71

&: The CNV index was between CNequal and CNminus and was referred to as "polymorphic"

%^*^: CNequal but partial genes were in polymorphic regions

NA: no CNV segments overlapping with gene regions of effectors

^*^: 68% identity to 70-15 BAS1

^**^: 99% identity to 70-15 BAS1

^#^: B71 has an insertion on the gene
