# Supplementary material for: Effector gene reshuffling involves dispensable mini-chromosomes in the wheat blast fungus
Source: PLoS Genet. 2019 Sep 12;15(9):e1008272. doi: 10.1371/journal.pgen.1008272 (PMC6741851; doi:10.1371/journal.pgen.1008272)
Supplement: S4 Table — (DOCX) [file pgen.1008272.s018.docx]

**S4 Table.** Copy number of effectors based on assembled sequences

| Effector | B71^1^ | T25 | P3 | B2 | B51 | P28 | P29 | Py22.1 | Py5020 | 70-15^1^ |
| --- | --- | --- | --- | --- | --- | --- | --- | --- | --- | --- |
| PWT4 | Ø | § | Ø | Ø | Ø | Ø | § | Ø | Ø | Ø |
| PWT3 | IN | + | P | + | IN | + | IN | IN | IN | + |
| PWL4 | + | + | + | + | + | + | + | + | + | Ø |
| PWL3 | Ø | Ø | Ø | Ø | Ø | Ø | Ø | Ø | Ø | + |
| PWL2 | + | Ø | + | Ø | Ø | + | Ø | Ø | + | ++ |
| PWL1 | Ø | Ø | Ø | Ø | Ø | Ø | Ø | Ø | Ø | Ø |
| BAS4 | + | + | + | + | + | + | + | + | + | + |
| BAS3 | + | + | + | + | + | + | + | + | + | + |
| BAS2 | + | + | + | + | + | + | +^2^ | + | +^2^ | + |
| BAS1 | §+ | § | §+ | § | § | §+ | § | § | §+ | §+ |
| AVRPiz-t | + | + | + | + | + | + | + | + | + | + |
| AVR-Pita3 | + | + | + | + | + | + | Ø | + | + | + |
| AVR-Pita2 | Ø | Ø | Ø | Ø | Ø | Ø | Ø | Ø | Ø | Ø |
| AVR-Pita1 | Ø | Ø | Ø | Ø | Ø | Ø | Ø | Ø | Ø | + |
| AVR-Pik_km_kp | §§ | §§ | §§ | §§ | §§ | §§ | §§ | §§ | §§ | + |
| AVR-Pii | §§ | § | § | § | + | § | §§ | § | § | Ø |
| AVR-Pib | + | + | + | + | Ø | + | + | + | + | + |
| AVR-Pia | Ø | Ø | Ø | Ø | Ø | Ø | Ø | Ø | Ø | Ø |
| AVR-Pi54 | + | + | + | + | + | + | + | + | + | + |
| AVR-Pi9 | + | + | + | + | + | + | + | + | + | + |
| AVR-CO39 | IN | IN | IN | IN | IN | IN | IN | IN | IN | Ø |

^1^tblastn was used to search homologs (Evalue < 1e-30). The B71Ref1 and MG8 were used for B71 and 70-15, respectively. Assemblies from Illumina data were used for other strains.

^2^sequences are different with other BAS2

Ø: no homologs based on the Blast result

IN: inserted by other sequence;

+: one copy of highly similar sequence (>90%) as listed proteins;

++: two copies of highly similar sequence

§: one copy of moderately similar sequence (>50% but <90%) as listed proteins;

§§: two copies of moderately similar sequence

P: partial sequences identified in draft assemblies
