# Supplementary material for: Effector gene reshuffling involves dispensable mini-chromosomes in the wheat blast fungus
Source: PLoS Genet. 2019 Sep 12;15(9):e1008272. doi: 10.1371/journal.pgen.1008272 (PMC6741851; doi:10.1371/journal.pgen.1008272)
Supplement: S5 Table — (DOCX) [file pgen.1008272.s019.docx]

**S5 Table.** Summary of repetitive elements of the B71 MoT genome

| Repeats | Counts | Total (bp) / (% of genome) | Counts in core | Total (bp) in core / (% of core) | Counts in mini | Total in mini (bp) / (% of mini) |
| --- | --- | --- | --- | --- | --- | --- |
| Class I (LTR) |  |  |  |  |  |  |
| Copia | 698 | 554,832/1.25 | 594 | 496,082/1.17 | 104 | 58,750/3.04 |
| Gypsy | 1,322 | 1,678,110/3.77 | 1,040 | 1,411,961/3.32 | 282 | 266,149/13.79 |
| unknown | 692 | 626,450/1.41 | 590 | 537,936/1.26 | 102 | 88,514/4.59 |
| Class I (LINE) |  |  |  |  |  |  |
| Tad1 | 165 | 249,837/0.56 | 94 | 141,286/0.33 | 71 | 108,551/5.62 |
| CRE | 143 | 172,313/0.39 | 101 | 138,429/0.33 | 42 | 33,884/1.76 |
| Jockey | 85 | 65,307/0.15 | 55 | 35,249/0.08 | 30 | 30,058/1.56 |
| I | 92 | 22,508/0.05 | 59 | 8,882/0.02 | 33 | 13,626/0.71 |
| R2 | 26 | 14,163/0.03 | 22 | 13,661/0.03 | 4 | 502/0.03 |
| Class I (SINE) |  |  |  |  |  |  |
| Alu | 192 | 71,583/0.16 | 148 | 37,856/0.09 | 44 | 33,727/1.75 |
| Mg | 1 | 195/0.0004 | 0 | 0/0 | 1 | 195/0.0101 |
| Class II (DNA) |  |  |  |  |  |  |
| TcMar-Fot1 | 395 | 306,805/0.69 | 282 | 197,036/0.46 | 113 | 109,769/5.69 |
| TcMar-Pogo | 8 | 3,135/0.01 | 0 | 0,000/0.00 | 8 | 3,135/0.16 |
| MITE | 572 | 152,506/0.34 | 528 | 144,451/0.34 | 44 | 8,055/0.42 |
| Unclassified elements | 2,209 | 1,222,299/2.75 | 1,839 | 957,562/2.25 | 370 | 264,737/13.72 |
| ***Total transposon elements*** | 6,600 | 5,140,043/11.56 | 5,352 | 4,120,391/9.69 | 1,248 | 1,019,652/52.83 |
| rRNA | 25 | 46,010/0.10 | 25 | 46,010/0.11 | 0 | 0/0 |
| Satellite | 77 | 8,595/0.02 | 77 | 8,595/0.02 | 0 | 0/0 |
| Simple_repeat | 12,715 | 466,976/1.05 | 12,606 | 462,423/1.09 | 109 | 4,553/0.24 |
| Low_complexity | 1,667 | 73,416/0.17 | 1,654 | 72,867/0.17 | 13 | 549/0.03 |
| ***Total*** | 21,084 | 5,735,040/12.90 | 19,714 | 4,710,286/11.08 | 1,370 | 1,024,754/53.10 |
