# Supplementary material for: Effector gene reshuffling involves dispensable mini-chromosomes in the wheat blast fungus
Source: PLoS Genet. 2019 Sep 12;15(9):e1008272. doi: 10.1371/journal.pgen.1008272 (PMC6741851; doi:10.1371/journal.pgen.1008272)
Supplement: S6 Table — (DOCX) [file pgen.1008272.s020.docx]

**S6 Table.** List of primers or oligos used in this study

| Name | Sequence (5'-3') | Purpose; note |
| --- | --- | --- |
| L95 | CGGCCAGTGAATTGTAATACG | LIEP; 1^st^ PCR primer for linker sequencing |
| RPtag | GACTGGAGTTCAGACGTGTGCTCTTCCGATCTgagtc | LIEP; 1^st^ PCR primer for linker sequencing |
| htF501s2 | AATGATACGGCGACCACCGAGATCTACACTATAGCCTACACTCTTTCCCTACACGACGCTCTTCC | LIEP; 2^nd^ PCR primer for linker sequencing |
| htF701s | CAAGCAGAAGACGGCATACGAGATCGAGTAATGTGACTGGAGTTCAGACGTGTG | LIEP; 2^nd^ PCR primer for linker sequencing |
| hta1 | GACTGGAGTTCAGACGTGTGCTCTTCCGATCT | LIEP; oligo for making a Y-adaptor |
| hta2 | GATCGGAAGAGCACACaaa | LIEP; oligo for making a Y-adaptor |
| 501Tr1 | CTACACGACGCTCTTCCG | LIEP; 1^st^ PCR primer for clone sequencing library |
| 701Tr1 | GACTGGAGTTCAGACGTGTG | LIEP; 1^st^ PCR primer for clone sequencing library |
| htF501s | AATGATACGGCGACCACCGAGATCTACACTATAGCCTACACTCTTTCCCTACACGACGCTCTTCCGAT | LIEP; Illumina TruSeq HT primer used in 2^nd^ PCR for clone sequencing library |
| htF502s | AATGATACGGCGACCACCGAGATCTACACATAGAGGCACACTCTTTCCCTACACGACGCTCTTCCGAT | LIEP; Illumina TruSeq HT primer used in 2^nd^ PCR for clone sequencing library |
| htF702s | CAAGCAGAAGACGGCATACGAGATTCTCCGGAGTGACTGGAGTTCAGACGTGTG | LIEP; Illumina TruSeq HT primer used in 2^nd^ PCR for clone sequencing library |
| htF703s | CAAGCAGAAGACGGCATACGAGATAATGAGCGGTGACTGGAGTTCAGACGTGTG | LIEP; Illumina TruSeq HT primer used in 2^nd^ PCR for clone sequencing library |
| htF704s | CAAGCAGAAGACGGCATACGAGATGGAATCTCGTGACTGGAGTTCAGACGTGTG | LIEP; Illumina TruSeq HT primer used in 2^nd^ PCR for clone sequencing library |
| BAS1-F | GGTGCTTGCCACCTTTACC | genomic amplification; *BAS1* primer |
| BAS1-R | TTCTCCACCCGTCTAATACCA | genomic amplification; *BAS1* primer |
| PWL2-F | TCCTCCCTTTTGCTTTGTTC | genomic amplification; *PWL2* primer |
| PWL2-R | ATAATATCCGTCGCCCCATT | genomic amplification; *PWL2* primer |
| MgActinF | AGCGTGGTATCCTCACTTTGC | qRT-PCR; genomic amplification; *actin* primer |
| MgActinR | ATCTTCTCTCGGTTGGACTTGG | qRT-PCR; genomic amplification; *actin* primer |
| MgPwl2F | CCGCCGAATACGGAAATCAC | qRT-PCR; *PWL2* primer |
| Pwl2_qRT2-R4 | CAGCCCTCTTCTCGCTGTTC | qRT-PCR; genomic amplification; *PWL2* primer |
| MgBas1F | CGCGGCTGAAGATTATTCC | qRT-PCR; *BAS1* primer |
| MgBas1R | TTCTCCACCCGTCTAATACC | qRT-PCR; *BAS1* primer |
